# The binding pocket properties were fundamental to functional diversification of the GDSL-type esterases/lipases gene family in cotton

**DOI:** 10.3389/fpls.2022.1099673

**Published:** 2023-01-18

**Authors:** Jianshe Wang, Haiyan Zhao, Yunfang Qu, Peng Yang, Jinling Huang

**Affiliations:** ^1^ College of Agriculture, Shanxi Agricultural University, Taigu, Shanxi, China; ^2^ School of Biotechnology and Food Engineering, Anyang Institute of Technology, Anyang, Henan, China

**Keywords:** GDSL-type esterases/lipases, binding pocket, functional diversity, *Gossypium* species, molecular simulation, molecular mechanism

## Abstract

Cotton is one of the most important crops in the world. GDSL-type esterases/lipases (GELPs) are widely present in all kingdoms and play an essential role in regulating plant growth, development, and responses to abiotic and biotic stresses. However, the molecular mechanisms underlying this functional diversity remain unclear. Here, based on the identification of the *GELP* gene family, we applied genetic evolution and molecular simulation techniques to explore molecular mechanisms in cotton species. A total of 1502 *GELP* genes were identified in 10 cotton species. Segmental duplication and differences in evolutionary rates are the leading causes of the increase in the number and diversity of *GELP* genes during evolution for ecological adaptation. Structural analysis revealed that the GELP family has high structural diversity. Moreover, molecular simulation studies have demonstrated significant differences in the properties of the binding pockets among cotton GELPs. In the process of adapting to the environment, GELPs not only have segmental duplication but also have different evolutionary rates, resulting in gene diversity. This diversity leads to significant differences in the 3D structure and binding pocket properties and, finally, to functional diversity. These findings provide a reference for further functional analyses of plant GELPs.

## Introduction

GDSL-type esterases/lipases (GELPs), a lipid hydrolysis enzyme, typically have a Ser-His-Asp active site near the N-terminus (Upton and Buckley, 1995). Because of the four strictly conserved sites of Ser-Gly-Asn-His in four blocks of homology, a subfamily of the GDSL family has been further classified as SGNH-hydrolase (Upton and Buckley, 1995; Akoh et al., 2004). GELPs are widely present in all kingdoms and play a vital role for regulating plant growth and development, organ morphogenesis, secondary metabolism, plant immunity, and stress. *RMS2*, an endoplasmic reticulum-localized GDSL lipase gene, has a significant role in the male fertility regulatory network in rice (Zhao et al., 2020c). Additionally, to regulate root growth in rice, MHZ11 is involved in ethylene signalling (Zhao et al., 2020a). Three GELPs, OsGELP34, OsGELP110, and OsGELP115, are required for the development of rice pollen (Zhang et al., 2020). Many GELPs have been reported to be involved in immunity in plants such as *Arabidopsis* and rice (Oh et al., 2005; Lee et al., 2009; Gao et al., 2017; Smyth, 2017). A study on SFARs showed that it could reduce fatty acid storage and change the composition of unsaturated fatty acids in *Arabidopsis* seeds (Huang et al., 2015). GDSL esterase is involved in xylan deacetylation for secondary wall patterning (Zhang et al., 2017), and overexpression of *AtLTL1* could improve salt stress tolerance in transgenic *Arabidopsis* plants (Naranjo et al., 2006). Moreover, the GDSL-type lipase gene showed drought tolerance in transgenic pepper plants (Hong et al., 2008).

The GELP family has attracted considerable attention because of its functional diversity. In recent years, an increasing number of GELP families have been identified and characterized in various plant species. For instance, a large family of GELPs have been identified and characterized in *Arabidopsis* (Lai et al., 2017), *Glycine max* (Su et al., 2020), *Vitis vinifera* L. (Ni et al., 2020), *Citrullus lanatus* (Ren et al., 2021), *Sedum alfredii* (Li et al., 2019), *Brassica rapa* (Dong et al., 2016), Rosaceae (Cao et al., 2018), *Oryza sativa* (Chepyshko et al., 2012), *Nicotiana tabacum* (Lv et al., 2021), *Dasypyrum villosum* (Zhang et al., 2021), *Pyrus* spp. (Zhang et al., 2022), *Solanum lycopersicum* (Sun et al., 2022), *Sorghum bicolor*, *Picea sitchensis*, *Populus trichocarpa*, and *Physcomitrella patens* (Volokita et al., 2011). However, the molecular mechanisms underlying this functional diversity have not yet been elucidated.

Cotton is one of the most important economic crops in the world and is the primary natural fibre material used in the textile industry. GELP studies have focused mainly on fibre and seed development (Tong et al., 2013; Yadav et al., 2017; Ma et al., 2018), lipid metabolism (Hao et al., 2014), and disease resistance (An et al., 2017). Fortunately, genomes of multiple cotton species have been sequenced (https://www.cottongen.org/). Although genome-wide identification of *GELP* genes in cotton has been performed, it has been limited to those in *Gossypium hirsutum*, unlike the systematic analysis of 10 cotton species [five diploid species (A genome, D genome) and five allotetraploid species (At/Dt)] performed in this study for evolutionary and functional aspects (Mo et al., 2022). Therefore, based on genome-wide identification of *GELP* genes, the purpose of our study was to analyse the evolution of *GELP* genes in 10 cotton species and to examine the molecular mechanism of functional diversity using molecular simulation.

## Materials and methods

### Plant materials, strains, and plasmids

In this study, the sea island cotton cultivars PS-7, Yuehai 9, and *Arabidopsis thaliana*, which is the wild-type and also of the Columbia ecotype (Col-0) background, were used. A *Trans*1-T1 phage-resistant chemically competent cells were purchased from TransGen Biotech (Beijing, China). The vectors pEASY-T3 and pRI 101-AN were used for cloning and expression, respectively.

### Identification of the GDSL-type esterase/lipase gene in cotton

In the present study, we identified *GELP* candidate genes in 10 *Gossypium* species: *G. herbaceum* (A_1_) (Huang et al., 2020), *G. arboreum* (A_2_) (Huang et al., 2020), *G. thurberi* (D_1_) (Grover et al., 2019), *G. raimondii* (D_5_) (Udall et al., 2019), *G. turneri* (D_10_) (Udall et al., 2019), *G. hirsutum* (AD)_1_ (Chen et al., 2020b), *G. barbadense* (AD)_2_ (Chen et al., 2020b), *G. tomentosum* (AD)_3_ (Chen et al., 2020b), *G. mustelinum* (AD)_4_ (Chen et al., 2020b), and *G. darwinii* (AD)_5_ (Chen et al., 2020b). The protein databases were obtained from CottonGen (Yu et al., 2021). The GELPs in *A. thaliana* were downloaded from TAIR (https://www.arabidopsis.org) and have been described in a previous report (Lai et al., 2017).

To identify the cotton *GELP* genes, the Hidden Markov model (HMM) profiles of the GDSL conserved domain (PF13472, PF14606, PF16255, and PF00657) from the Pfam database (http://pfam.xfam.org/) were used to scan the local database. Candidate GELPs were further verified using SMART (http://smart.embl-heidelberg.de/) and CDD (https://www.ncbi.nlm.nih.gov/Structure/bwrpsb/bwrpsb.cgi) online tools. Genes lacking complete GDSL-type esterase/lipase domains were excluded, splice variants were discarded, and only the first variant was retained for further analysis.

Physicochemical properties, including protein molecular weight (MW), instability index (II), and theoretical isoelectric point (pI), were predicted using the ExPASy server (https://web.expasy.org/protparam/). The WoLF PSORT program (https://wolfpsort.hgc.jp/) was used to predict protein subcellular localization (Horton et al., 2007), and transmembrane domains were analysed using TMHMM 2.0 (https://services.healthtech.dtu.dk/service.php?TMHMM-2.0). Signal peptides were identified using SignalP 5.0 (https://services.healthtech.dtu.dk/service.php?SignalP-5.0).

### Phylogenetic analysis and conserved motif analysis

The cotton GELP sequences from the 10 species and selected AtGELP protein sequences were aligned using MUSCLE in MEGA-X (Kumar et al., 2018). In this study, the ModelFinder software was used to determine the best-fit model for all GELPs (Kalyaanamoorthy et al., 2017). Thereafter, according to the Bayesian information criterion (BIC), VT+R10, as the best-fit model, was used in the phylogenetic tree of cotton and *Arabidopsis*. IQ-TREE2 was used to build an unrooted phylogenetic tree with ultrafast bootstraps, as well as the Shimodaira-Hasegawa approximate likelihood ratio test (SH-aLRT) (1000 replicates each) (Minh et al., 2020). iTOL tools (https://itol.embl.de/) were used to modify the phylogenetic tree (Letunic and Bork, 2019).

MEME software v5.3.0 (http://meme-suite.org/) was used to identify conserved motifs in the GDSL-type esterase/lipase proteins. Default parameters were used, with the exception that the maximum E-value of the motifs was set to 0.01.

### Chromosomal location and gene duplication

The chromosomal locations of the *GELP* genes were obtained from genome annotation files. Gene duplication events were obtained using the Multiple Collinearity Scan Toolkit (MCScanX) (Wang et al., 2012). Ka, Ks, and Ka/Ks values were calculated using the TBtools software (Chen et al., 2020a).

### Identification of orthologous *GELP* genes

Orthologous proteins from ten cotton species were identified using OrthoVenn2 (Xu et al., 2019). All the *GELP* genes from these cotton species and *A. thaliana* were used in the analysis. To identify orthologous gene clusters, each cotton species was individually assessed and compared with each other, as well as with *Arabidopsis*.

### *Cis*-element analysis

The 2 kb sequences upstream from the translational start sites of all *GELP* genes were obtained by searching the cotton genome database, and the online website PlantCARE (http://bioinformatics.psb.ugent.be/webtools/plantcare/html/) was used to predict *cis*-regulatory elements.

### GELPs 3D structure analysis

To obtain information about the tertiary structure of GELPs, based on the phylogenetic tree, 16 protein structures were computed using two prediction methods: AlphaFold2 (Jumper et al., 2021) and homology modelling using Discovery Studio software (BIOVIA, San Diego, CA, USA). The quality of the theoretical model was evaluated using the Profiles-3D program. Structural similarity was analysed using the 3DMA program.

### Profiling of *GELPs* substrate specificity

To better understand the function of cotton GELPs, we performed molecular docking using Discovery Studio software for substrate specificity. The lipid structures were obtained from the LIPID MAPS^®^ Structure Database (https://www.lipidmaps.org/). CDOCKER is a grid-based molecular docking method that uses CHARMm for molecular docking. Before starting the docking process, the 3D structures were optimized using the Prepare Protein protocol. After all ligands were minimized in the receptor molecule *via* the *in situ* ligand minimization program, docking between the minimized ligands and the receptor was performed using the CDOCKER program in the default mode. The results of molecular docking were evaluated based on the –CDOCKER_INTERACTION_ENERGY score and the non-bonding interactions of three conserved catalytic residues. Molecular dynamics were analysed using the Discovery Studio software.

### Gene expression profile analysis

Gene expression profiling provides vital evidence for investigating the biological functions of the *GELP* genes. To analyse the expression patterns of cotton *GELPs* under different conditions, including growth and development, various biotic and abiotic stresses and the transcriptome data (accession: PRJNA248163, PRJNA275482, PRJNA576973) were used to examine the relative expression patterns of cotton *GELPs*. Abiotic stress consisted of cold, hot, salt, and polyethylene glycol (PEG) exposure at various time intervals (0, 1, 3, 6, and 12 h for each treatment). The different tissues included roots, stems, leaves, thorns, petals, stamens, pistils, calyces, ovules, and fibres. The biotic stresses on cotton aphids and *Aspergillus flavus* were also investigated. Several time points were assessed after cotton aphid infestation (0, 6, 12, 24, 48, and 72 h). A heat map was generated for relative expression analysis using TBtools software, which was normalized by Log_2_ (FPKM values) values, followed by hierarchical clustering.

### Quantitative real-time PCR analysis of *GbaGELP142D* in sea island cotton

The sea island cotton materials Yuehai 9 and PS-7 were cultured using Hoagland nutrient solution hydroponics. The hydroponic seedlings were divided into two parts and treated with NaCl for short-term stress and long-term stress.

Short-term stress: When cotton seedlings grow to two leaves and one heart, seedlings with consistent growth are selected and divided into five groups, with three biological repeats set for each group. The treatment group was treated with NaCl at a concentration of 300 mmol·L^-1^, while the control group was continuously treated with the same amount of Hoagland nutrient solution. The leaf and whole root samples of the treatment and control groups were collected at 0, 4, 8, 12, and 24 h, frozen in liquid nitrogen, and stored at -80°C until use.

Long-term stress: From germination, the treatment group was continuously treated with 150 mmol·L^-1^ NaCl, whereas the control group was continuously irrigated with the same amount of Hoagland nutrient solution. They were divided into two groups, and each group was set with three biological replicates. When cotton seedlings grew to two leaves and one heart, samples of leaves and whole roots in the treatment and control groups were collected, frozen in liquid nitrogen, and stored at -80°C until use.

Total RNAs extraction, reverse transcription, quantitative real-time PCR (qRT-PCR), and the relative expression of *GbaGELP142D* in the leaves and roots of Yuehai 9 and PS-7 were performed according to Zhao et al.’s method (2020b). The primers used for the qRT-PCR analysis are shown in **Supplementary Table S1**.

### Gene cloning

According to our transcriptomics (not published) of the sea-island cotton cultivars PS-7 and Yuehai 9 under salt stress, *GbGELP142D* was significantly upregulated in response to salt stress in both sea-island cotton cultivars. Total RNA from the leaves was extracted using the EASYspin Plus Plant RNA Kit RN37 (Aidlab Biotechnology) and cDNA synthesis was performed using the M-MLV RTase cDNA Synthesis Kit (TaKaRa Company). A pair of primers (**Table S1**.) targeting *GbGELP142D* was designed based on its open reading frame (ORF). The resulting amplicon was ligated into the pEASY-T3 vector and confirmed using DNA sequencing.

### Construction of the *GbGELP142D* overexpression vector and *Arabidopsis* transformation

The open reading frame of *GbGELP142D* was cloned into pRI 101-AN, and the vector was transferred into *Agrobacterium tumefaciens* strain GV3101. Transgenic *Arabidopsis* plants were created through *Agrobacterium*-mediated transformation using the floral dipping method (Clough and Bent, 1998). T_1_ and T_2_ seeds were screened on 50 mg/L kanamycin plates to select homozygous progenies. Based on the relative quantitative method of qRT-PCR technology (Livak and Schmittgen, 2001), the line with the lowest expression of *GbaGELP142D* was used as the control, and the three lines were selected as the research objects of salt tolerance in the later stage. The primers used for the qRT-PCR analysis are shown in **Supplementary Table S1**.

### Salt stress tolerance assay of *GbaGELP142D* overexpression in transgenic *Arabidopsis*

To assess the salt tolerance of the transgenic *Arabidopsis* plants, transgenic seeds and the wild-types were grown on MS plates which containing 0 mM NaCl and 120 mM NaCl respectively, after three days of vernalization, the germination rate was investigated for 11 consecutive days. These plants were photographed, and their status was observed 30 days after sowing.

*Arabidopsis* seedlings were grown on MS plates containing 0 mM NaCl for 10 d and were transplanted into sterile vermiculite. After 15 d of normal culture, *Arabidopsis* seedlings were irrigated with 0 mM NaCl and 120 mM NaCl. After seven days of treatment, plant height, fresh weight above ground, root length, and chlorophyll content (Zou, 1995) were measured. Four biological replicates, with four technical replicates per sample, were used.

## Results

### Identification and characterization of the *GELP* genes in the 10 cotton species (A genome, D genome, and At/Dt)

**Figure 1:**
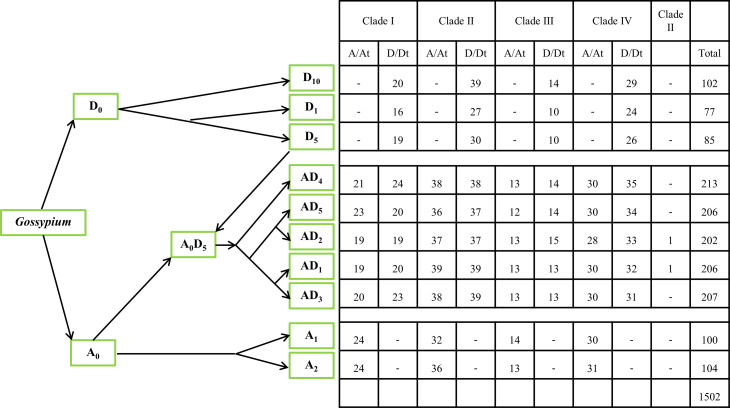
The phylogeny of the 10 cotton species and the number of *GELP* genes used in this study. The phylogeny of the 10 cotton species (A genome, D genome, and At/Dt) was constructed based on data from previous studies (Grover et al., 2019; Chen, ZJ et al., 2020b; Huang, G et al., 2021). - represents the absence of gene at this branch.

A total of 1502 *GELP* genes encoding the GDSL domain were identified in ten cotton species: *G. herbaceum*, *G. arboreum*, *G. thurberi*, *G. raimondii*, *G. turneri*, *G. hirsutum*, *G. barbadense*, *G. tomentosum*, *G. mustelinum*, and *G. darwinii* (**Figure 1**). Ghe, Gar, Gth, Gra, Gtu, Ghi, Gba, Gto, Gmu, Gda, and At were used as prefixes before the names of the *GELP* genes for the five diploid species (A and D genomes), five allotetraploid species (At/Dt), and *A*. *thaliana*, respectively. A and D were used as postfixes after the names of *GELP* genes for the A genome or At sub-genomes, and D genome or Dt sub-genomes, respectively.

Based on their chromosomal location, the *GELPs* from these 10 species were renamed *GarGELP1A*-*GarGELP104A*, *GbaGELP1A*-*GbaGELP202*, *GdaGELP1A*-*GdaGELP206D*, *GheGELP1A*-*GheGELP100A*, *GhiGELP1A*-*GhiGELP206*, *GmuGELP1A*-*GmuGELP213D*, *GraGELP1D*-*GraGELP85D*, *GthGELP1D*-*GthGELP77D*, *GtoGELP1A*-*GtoGELP207D*, and *GtuGELP1D*-*GtuGELP102D* (**Table S2**). In total, 100–104 *GELP* genes were identified in A genomes, 77–102 *GELP* genes were identified in D genomes, and 202–213 *GELP* genes were identified in the AtDt genomes.

The gene names, gene IDs, number of amino acids, theoretical pI, molecular weight, the total number of negatively charged residues (Asp+Glu), the total number of positively charged residues (Arg+Lys), instability index, whether they are stable/unstable, grand average of hydropathicity (GRAVY), aliphatic index, signal peptide prediction, number of predicted TMHs, subcellular localization, and chromosome locations are listed in **Table S2**. The lengths of the GELPs ranged from 193 to 1224 amino acid residues, with an average sequence length of 367 amino acids. The molecular weights ranged from 21.38 to 134.83 kDa, averaging 40.63 kDa. The pI varied from 4.65 to 9.64, with an average of 7.11. The number of negatively charged residues ranged from 16 to 157 and the number of positively charged residues ranged from 16 to 155. According to the instability index, up to 86.7% (1302 of 1502) of the proteins identified were stable. The aliphatic index ranged from 63.19 to 108.36, with a mean of 85.98. The grand average hydropathicity ranged from -0.677 to 0.29, with an average of -0.07, and the basic physiochemical properties of GELPs showed differences among the cotton species (**Table S2**).

The subcellular localization prediction showed that the identified GELPs were located in all parts of the cell. In each cotton species, >64% GELPs contained signal peptides, >29% GELPs contained one potential transmembrane-spanning domain, and 1–4 GELPs had two potential transmembrane-spanning domains in each cotton species (**Table S2**).

### Phylogenetic analysis and conserved motif analysis

**Figure 2:**
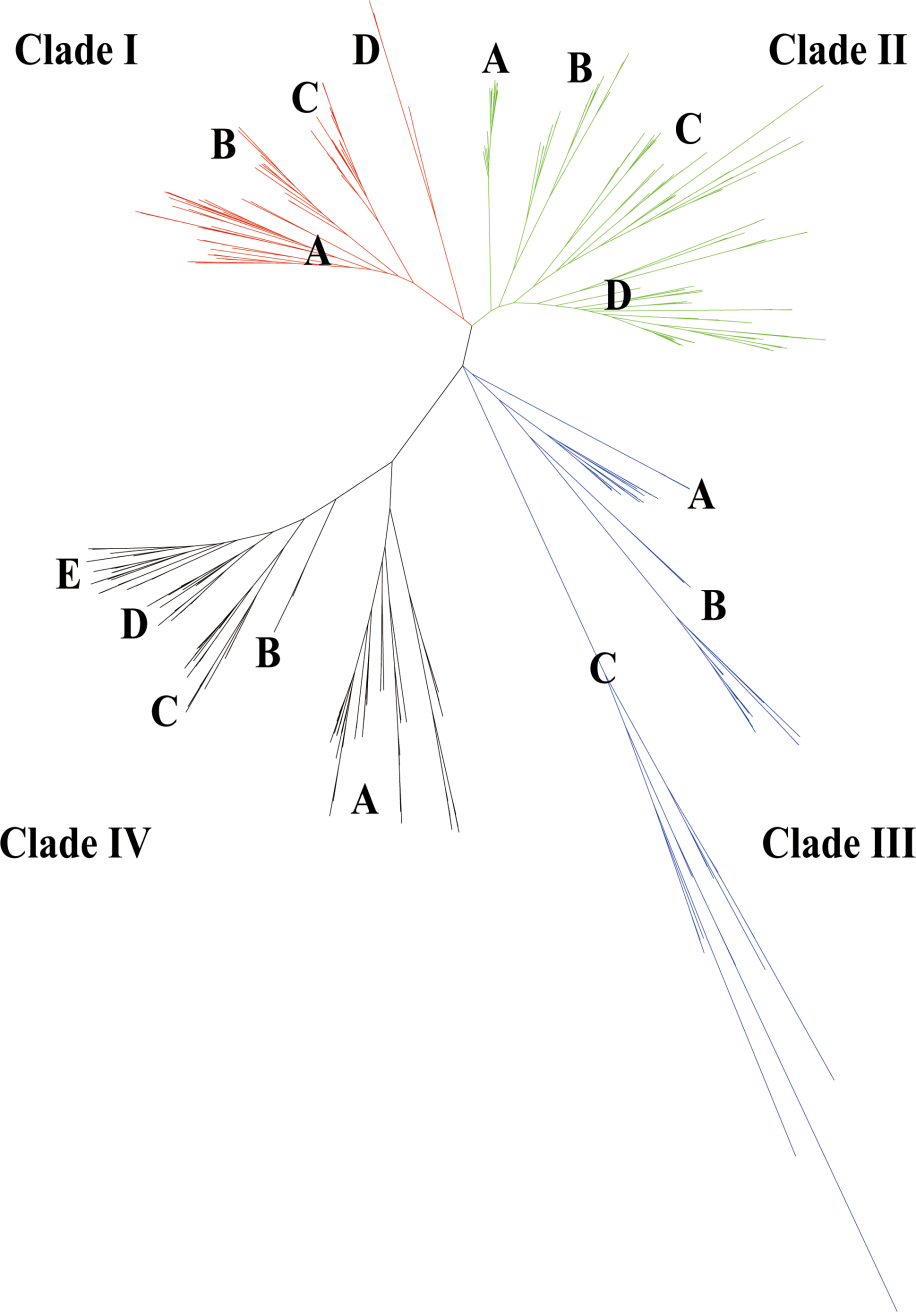
Evolutionary tree of the GELP family in the 10 cotton species (A genome, D genome, and At/Dt) and in *Arabidopsis thaliana*. A total of 1502 GELPs could be classified into four clades (I–IV) indicated with four branch colours. Every clade had 3-5 subclades (such as, A, B, C, D and E).

To investigate the evolution of *GELP* genes and their phylogenetic relationships in the 10 cotton species and *Arabidopsis*, IQ-TREE2 was used to build an unrooted phylogenetic tree. Consistent with previous studies on *Arabidopsis* (Lai et al., 2017), these GELPs were classified into four clades (I–IV). Clades I, II, III, and IV comprised 311, 544, 194, and 453 *GELPs*, respectively, and were further divided into four, four, three, and five subclades, respectively (**Figure 2**; **Table S3**). AtGELP62, AtGELP85, and AtGELP86 were placed in clade III. We also found that the number of *GELP* genes derived from the Dt sub-genome of the five allotetraploid cotton species was greater than that of the *GELP* genes of the D_5_ genome.

To investigate *GELP* gene diversification in cotton, conserved protein motifs were analysed using the MEME online server. Conserved motifs represent essential sites for enzymatic function. A total of 140 conserved motifs were identified (E values<0.01) in the 1502 GELPs. Conserved motifs (4, 6, 9, and 3) of typical GDSL esterase/lipase proteins (blocks I, II, III, and V, respectively) were revealed, except motifs 36 and 71 for block I in subclade IIIC.

All four clades contained 62 of 140 motifs. Furthermore, each clade (except clade I) contained specific motifs that represented its own subclade functions (**Figure S1**, **Table S4**). For example, motifs 32, 35, 40, 49, 69, 79, 88, 131, and 136 were only detected in clade II, Clade III had seven particular motifs (motifs 21, 22, 29, 30, 31, 42, and 53), and motifs 20, 23, 33, 55, 56, and 89 were only detected in clade IV.

### Chromosomal location and gene duplication

The *GELP* genes were unevenly distributed on every chromosome in cotton. However, there were only two *GELP* genes (*GbaGELP202* and *GhiGELP206*) in the scaffolds. Specifically, the *GELP* genes from the A_1_ and A_2_ genomes were mainly located on Chr A05, A07, A08, and A10; *GELP* genes from the D_1_, D_5_, and D_10_ genomes were primarily located on Chr D05, D07, D08, and D11; and *GELP* genes from five allotetraploid species were distributed mainly on Chr A05, A07, A08, A11, D05, D07, D08, D11, and D13 (**Figure S2**, **Table S2**).

The distribution of *GELP* genes over the At/Dt sub-genome was very similar across the five allotetraploid cotton species, and the distribution of *GELP* genes for the Dt and D_5_ genomes was also similar. Moreover, compared with the *GELP* distribution on the Dt sub-genome, the distribution patterns on the At sub-genome and A_1_/A_2_ genomes were less similar. This is because D_5_ is the D genome donor of allotetraploid cotton species and A_0_ is a common ancestor of A_1_, A_2_, and the A_t1_ sub-genome in the five allotetraploids (Huang et al., 2020).

The duplication of genes plays a vital role in the expansion of genes and production of new functions. In this study, to elucidate how *GELP* genes are duplicated, we investigated the different duplication modes between and within species. MCScanX software was used to detect collinearity alignments. The results indicated that many *GELP* genes were located within the syntenic blocks on chromosomes. Overall, we detected 10722 duplicate gene pairs among the 10 cotton species (**Table S5**). The number of duplicate gene pairs varies substantially between species. For example, only 23 segmental duplication events were identified between *G. thurberi* and *G. raimondii*, whereas 511 segmental duplication events were found between *G. tomentosum* and *G. mustelinum*. There was also a significant variation in the number of gene duplications within the species (**Table S5**). In addition to *G. thurberi*, segmental and tandem duplications were found in nine cotton species.

Synonymous substitutions (Ks), non-synonymous substitutions (Ka), and Ka/Ks were calculated for the selected types of duplicated *GELP* genes. Generally, Ka/Ks = 1 indicates neutral selection, Ka/Ks < 1 indicates purifying selection, and Ka/Ks > 1 indicates accelerated evolution with positive selection. Gene duplication between A genome and A_t1_ sub-genome in the five allotetraploids showed that the Ka/Ks ratios of 69.3–74.3% of the orthologous pairs were below 0.5, while 13.7–20.0% of the pairs ranged from 0.5 to 1. We also noted that the Ka/Ks ratios for 60–83.6% of the syntenic paralogs were below 0.5, while 11.1–25.5% of the pairs ranged from 0.5 to 1 (**Table S5**). Based on these results, we reasoned that the cotton *GELP* gene family underwent intense purifying selection pressure, with limited functional divergence occurring after segmental and whole genome duplication (WGD).

Gene duplication analysis showed that 69 *GELP* genes derived from the D_5_ genome formed orthologous pairs with all five Dt sub-genomes, 74 *GELP* genes derived from the A_1_-genome formed orthologous pairs with all five At sub-genomes, and 84 *GELP* genes derived from the A_2_ genome formed orthologous pairs with all five At sub-genomes. Gene duplication between the A/D and A_t_/D_t_ sub-genomes in the five allotetraploids revealed that the Ka/Ks ratios of orthologous pairs were different among the five allotetraploids (**Table S6**). These findings indicate that *GELP* genes have different rates of evolution among the five allotetraploids.

### Identification of orthologous gene clusters

**Figure 3:**
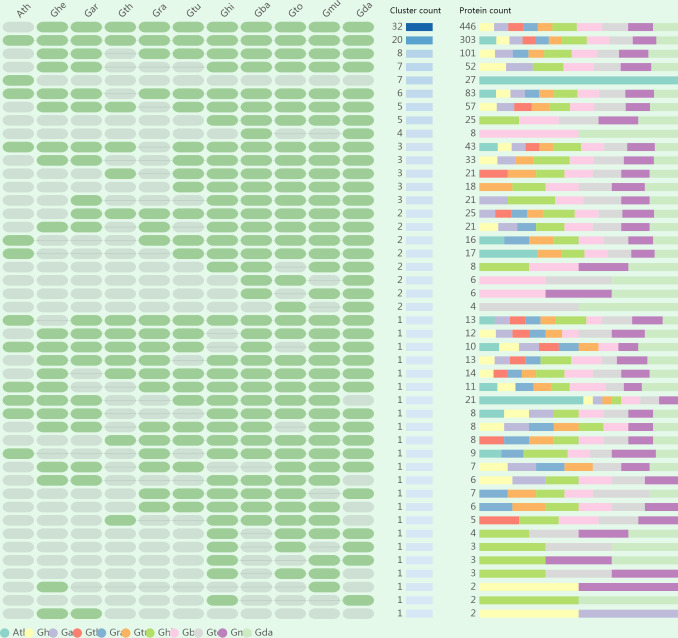
The occurrence pattern of shared orthologous groups among 10 cotton species and *Arabidopsis thaliana*.

To assess polyploidization events in the evolution of *GELP* genes, we identified specific and common orthologous clusters across 11 species (10 cotton species and *A. thaliana*). The identified orthologous gene clusters and their overlapping regions in the 11 species are shown in **Figure 3**; **Figure S3**, and **Figure S4**. A total of 148 orthologous gene clusters were obtained in 11 species. Among them, 39 common orthologous gene clusters were found in cotton and *A. thaliana*. In addition, there were 102 orthologous gene clusters which were solely composed of genes found in the cotton species, whereas *A. thaliana* had seven exclusive orthologous gene clusters. Most clusters were recorded from (AD)_5_, followed by (AD)_2_, (AD)_4_, (AD)_3_, (AD)_1_, A_2_, D_10_, A_1_, D_5_, and D_1_. These results revealed that polyploidization resulted in the evolution of new cotton-specific orthologous gene clusters.

We also constructed orthologous gene clusters between each cotton species as well as with *A. thaliana* separately. Between 1144 and 1176 orthologous genes were identified in the five allopolyploid species, 843, 864, 670, 745, and 858 orthologous genes were identified in A_1_, A_2_, D_1_, D_5_, and D_10_, respectively, whereas 354 orthologous genes were identified in *A. thaliana* (**Table S7**). Two in-paralogs were detected in D_1_, (AD)_1_, and (AD)_4_; four in-paralogs were detected in (AD)_5_; 234 in-paralogs were found in *A. thaliana*; but no in-paralogs were detected in other species. Between 11 and 14 singletons were identified in the five allopolyploid species, and 1 to 3 singletons were observed in the A and D genomes. The findings mentioned above indicate that the *GELP* gene family diverged across different species during evolution and was more likely to perform distinct functions.

### *Cis*-element analysis

The transcription level of a gene is controlled by *cis*-elements in conjunction with transcription factors. To explore the possible roles of GELPs in plant hormones, stress stimuli, and development, PlantCARE was used to analyse putative *cis*-elements in the promoter region (2.0 kb upstream ATG). This study investigated numerous putative cis-acting elements (71 types). Of these, five abiotic stress-responsive elements (ARE, DRE, LTR, MBS, and TC-rich repeats), 13 hormone-responsive elements (AuxRE/AuxRE-core/TGA-box/TGA-element, ABRE, ERE, GARE-motif/P-box/TATC-box, CGTCA-motif/TGACG-motif, and SARE/TCA-element), cell cycle regulation (MSA-like), circadian control (circadian), differentiation of the palisade mesophyll cells (HD-Zip 1), endosperm expression (GCN4_motif/AACA_motif), flavonoid biosynthetic genes regulation (MBSI), meristem expression (CAT-box), root-specific (motif I), phytochrome down-regulation expression (Unnamed_1), seed-specific regulation (RY-element), wounding and pathogen responsive (W box/WUN-motif), MYB, MYC, and light-responsive *cis*-elements. Overall, *cis*-elements related to abiotic stress, hormones, light-response, growth, and development were widely present in the promoters of *GELP* genes in the 10 cotton species. These results suggest that these *GELP* genes play essential roles in regulating growth and development, as well as in regulating responses to different sources of stress in cotton.

### 3D structure analyses of GELPs

Understanding the 3D structure of a protein is essential for understanding its function. GELPs of each subclade were used to find the most similar homology in the Protein Data Bank (PDB) database using the BLAST algorithm (https://www.rcsb.org/). Sixteen 3D structures of GELPs from each subclade were predicted using the homology modelling method. According to the primary sequence identity and crystal resolution models, 1–3 template crystal structures with six templates (6JKZ, 6UQV, 5XTU, 3MIL, and 1YZF) were used for homology modelling in this study for the 16 GELPs (**Table S8**). GDSL lipases have five conserved domains and four amino acid residues (Ser, Gly, Asn, and His), which play essential roles in enzyme catalysis. To improve the accuracy of homology modelling, the fixed blocks of the catalytic sites (GDSL, Asn, and His) were moved together to retain the alignment. To further improve the accuracy, structural models of the 16 GELPs were generated using AlphaFold2. Structural similarity was determined using the 3DMA program to compare the two prediction methods. Structural similarity analysis revealed that the backbone atom RMSD values of the two predicted structures were equal to or less than 1.6 Å. The results of Verify Protein (Profiles-3D) suggested that all predicted tertiary structure models of GELPs were reliable (**Table S8**).

**Figure 4:**
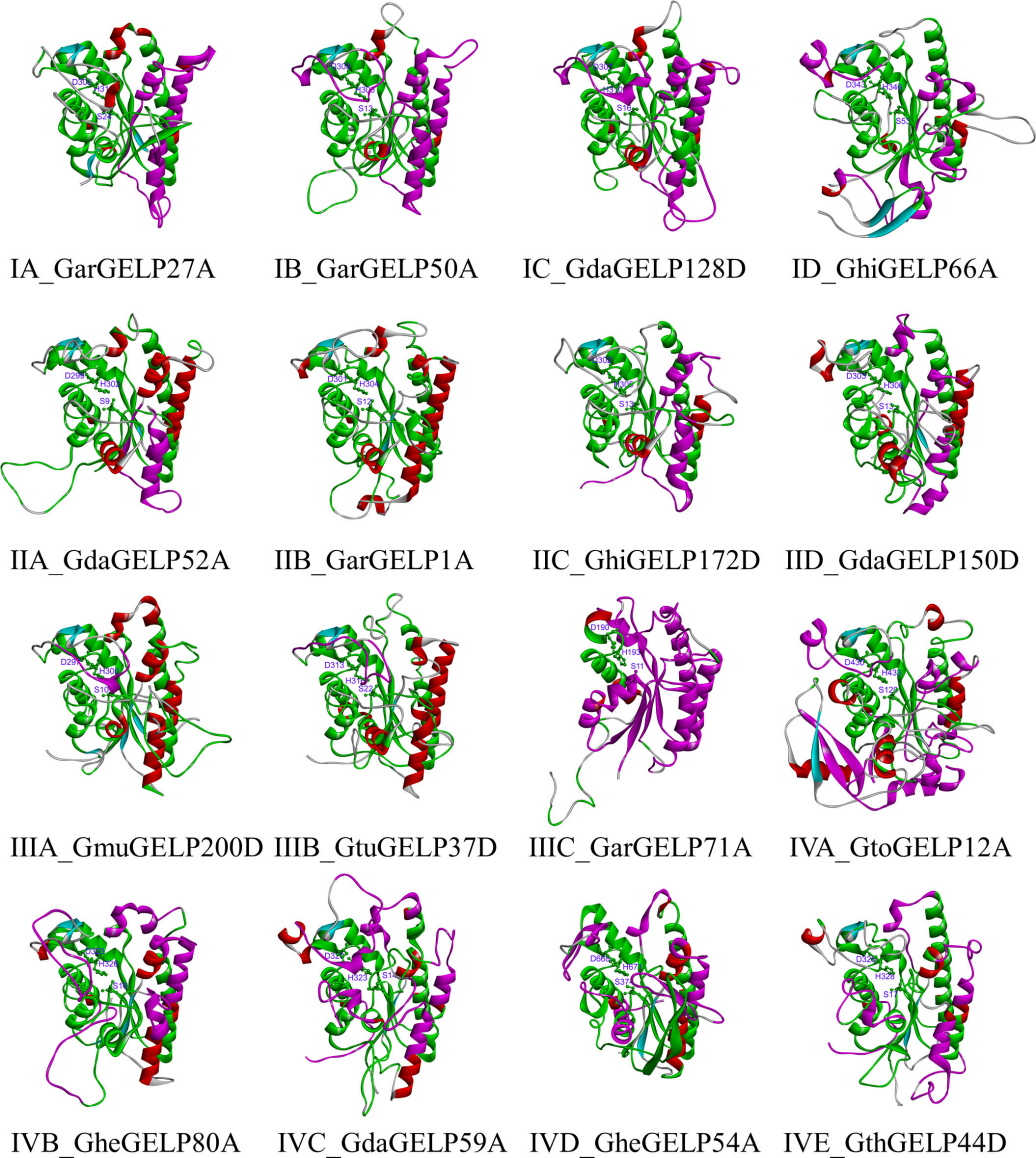
The tertiary structure of representative GDSL lipases belonging to subclades in the evolutionary tree. Green, co-occurrence motifs of four clades; Violet motifs specific to each clade. The catalytic triad Ser, Asp, and His are shown as sticks. The names of the tertiary structure of representative GDSL lipases consist of two parts: the subclade of the evolutionary tree and the gene name.

The 3D structures of GELPs showed that cotton GELPs are composed of several α-helices and β-folds, which are spherical structures. They are also composed of three conserved catalytic residues and are α/β-hydrolase foldable proteins (**Figure 4**). The secondary structure of the 16 proteins consisted of an α-helix, β-sheet, coil, and turn. In addition, GELP contain various secondary structures. Sixty-two motifs shared by the four clades were found at the centre of the protein. In addition, we found unique motifs mainly distributed on the surface and one β-fold in the centre. GarGELP71A, belonging to subclade IIIC, showed more notable differences in the protein’s advanced structure in the phylogenetic tree.

### Profiling of substrate specificity in GELPs

As one of the most widely used virtual screening methods, molecular docking can be used to predict the binding configurations of small molecules to protein receptors. Therefore, this is an effective strategy for the analysis of substrate specificity. Detailed analysis of *GELPs* substrate specificity is essential for identifying natural substrates and revealing their biological functions. The chemical structures of 46430 unique lipids were downloaded from the LIPID MAPS^®^ structure database. The lipids had eight categories: fatty acyls, glycerolipids, glycerophospholipids, sphingolipids, sterol lipids, prenol lipids, saccharolipids, and polyketides. Based on the 46430 unique lipids, 16 proteins from each subclade were used for these comparative studies through molecular docking. Three conserved catalytic residues (H, D, and S) were used to determine the active sites of GELPs. From the molecular docking results, it can be inferred that the larger values of –CDOCKER_INTERACTION_ENERGY represent a better affinity of the derivative for GELPs. Ligands with –CDOCKER_INTERACTION_ENERGY values greater than 0 were used to analyse the substrate specificity of the GELPs. Molecular docking screens indicated that the 16 GELPs contained 2526 ligands and 797 unique lipids. These lipids included 17 classes, including fatty esters, fatty acids and conjugates, fatty aldehydes, oxygenated hydrocarbons, and fatty alcohols, of which numbers were greater than 7%. We also found that GdaGELP128D in clade I contained 40 unique lipids, whereas GdaGELP59A in clade IV had 490 ligands. After docking, GhiGELP172D of clade IIC had zero candidate molecules with docking scores above 0. The ligands docked to the GELPs were mostly fatty acyls, such as (3Z)-buta-1,3-diene-1,1,4-tricarboxylic acid, 10-hydroxy-2Z,8Z-Decadiene-4,6-diynoic acid, 6-hydroxy-2-hexynoic acid, iodoethanoic acid, and 3-hexynoic acid. All four clades collectively had 136 unique lipids, and each clade contained specific lipids (**Figure S5**). For example, clades I, II, III, and IV contained 105, 21, 50, and 186 unique lipids, respectively. There were significant differences in lipids, even within the same clade of the evolutionary tree (**Table S9**).

**Figure 5:**
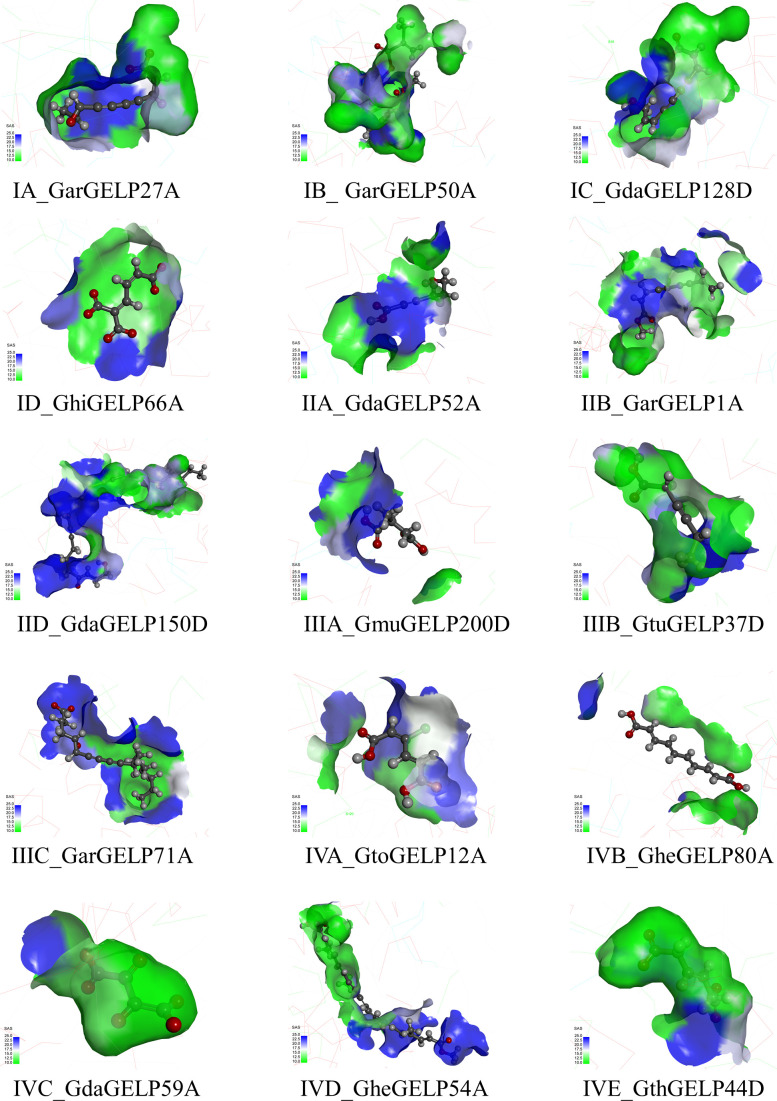
The binding pocket structure of GELPs. SAS: Creates a surface coloured by the solvent accessibility of the receptor residues from blue for ‘exposed’ to green for ‘buried’. The names of the binding pocket structures of representative GDSL lipases consist of two parts: the subclade of the evolutionary tree and the gene name.

To further reveal the functional differences in the *GELP* gene family, the best ligand molecules with maximum docking scores were selected to analyse the binding pocket and non-bonded interactions of the ligand-receptor complex. A binding pocket refers to a cavity on the surface or in the interior of a protein that possesses properties suitable for ligand binding. Although the active site residues of the 15 GELPs were identical, the binding pockets were different owing to their low structural similarity. This difference is manifested in shape, known as the solvent-accessible surface (SAS) (**Figure 5**). In addition, various non-bonding interactions exist in the complex of the 15 GELPs with lipids (**Figure S6**). These non-bonded interactions include hydrophobic interactions, hydrogen bonding, electrostatic interactions, and van der Waals forces. These results show that the main reason for the broad substrate spectrum is the properties of the GELPs’ binding pockets.

### Expression patterns of *GELP* genes in cotton

**Figure 6:**
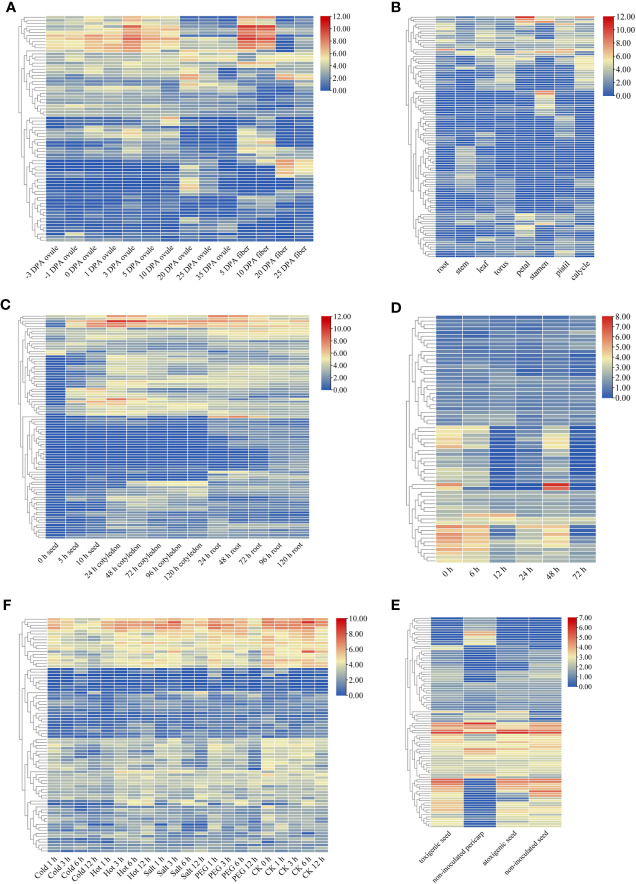
Expression patterns of *GELP* genes. Heatmap of the differential expression of *GELP* genes involved in the growth and development of ovule and fibre **(A)**; eight tissues (root, stem, leaf, torus, petal, stamen, pistil and calycle) **(B)**; the growth and development of seed, cotyledon, and root **(C)**. Heatmap of differential expression of GELP genes in response to stress, including cotton aphid attack (0 h, 6 h, 12 h, 24 h, 48 h, and 72 h) **(D)**, four abiotic stresses (cold, hot, salt and PEG) **(E)**, and atoxigenic and toxigenic strains of *Aspergillus flavus* **(F)**. The colour bar indicates log_2_ expression levels.

The raw data of the cotton plants were analysed in the present study to examine the variations in expression among the *GELP* genes across tissues. The results revealed that the expression of many *GELP* genes varied significantly among different tissues. For example, *Gh_A05G1329* and *Gh_D10G0307* showed significantly upregulated expression at 3 DPA and 5 DPA in the ovule, and 5 DPA and 10 DPA in the fibre (**Figure 6A**; **Table S10**). *Gh_Sca009545G02*, *Gh_A01G0634*, and *Gh_D08G2303* exhibited maximum expression in petals when compared to that in other tissues (**Figure 6B**; **Table S10**). The maximum expression of *Gh_A07G2062* and *Gh_D07G2278* was observed after 24 h in roots (**Figure 6C**; **Table S10**). *Gh_D06G2129* and *Gh_D05G2593* were expressed in the seed, cotyledon, and root, but much higher expression levels were found 10 h after seed germination and 24 h after germination in the cotyledon (**Figure 6C**; **Table S10**).

After the cotton aphid attack, the expression of many *GELP* genes in cotton leaves (*Ghir_A04G001760*, *Ghir_D01G007820*, and *Ghir_D10G006980*) decreased with increasing treatment time. However, only a few genes (*Ghir_D08G023300*) exhibited elevated expression levels (**Figure 6D**; **Table S10**). Expression analysis revealed that most of the *GELP* genes were differentially expressed in the toxigenic seed groups (**Figure 6E**; **Table S10**).

Abiotic stress, which includes cold, hot, salt, and drought stress, restricts the productivity and quality of cotton crops. These results suggest that the expression of multiple *GELP* genes can be induced by various abiotic stressors (**Figure 6F**; **Table S10**). Furthermore, hierarchical clustering patterns demonstrated that these *GELP* genes share similar gene expression profiles.

Moreover, the expression of *Gh_D06G1406* was significantly different under different abiotic stresses (cold, hot, PEG, and salt). In addition, *Ghir_D06G014530* was differentially expressed under biotic stresses (cotton aphid and aflatoxin from the fungus *A. flavus*). After analysing the data, the orthologous genes of *GbaGELP142D* are *Gh_D06G1406* and *Ghir_D06G014530* in *G. hirsutum*. From these results, it can be speculated that *GbaGELP142D* plays an important role in salt stress resistance.

### QRT-PCR analysis of *GbaGELP142D* in sea island cotton

The qRT-PCR analysis of *GbaGELP142D* was performed in the sea island cotton materials Yuehai 9 and PS-7, which were treated with NaCl short-term stress and long-term stress, respectively. It can be observed in **Figure S7**, at 4, 8 and 12 h of NaCl short-term stress, the relative expression of *GbaGELP142D* in the roots and leaves of Yuehai 9 was not significantly different. However, after 24 h of NaCl treatment, the expression of *GbaGELP142D* in the leaves of Yuehai 9 increased 27.98 times compared to that in the roots. It can be observed in **Figure S8**, compared with the 8, 12 and 24 h of NaCl short-term stress, at 4 h of NaCl stress, the relative expression of *GbaGELP142D* in the roots and leaves of PS-7 was the largest and the difference was significant, and the expression of *GbaGELP142D* in roots was 7.19 times than that in leaves. With the extension of NaCl stress time, the expression of *GbaGELP142D* in the roots and leaves of PS-7 decreased rapidly, reaching a minimum at 8 and 12 h of NaCl stress, respectively.

In terms of NaCl long-term stress, the relative expression of *GbaGELP142D* in the leaves of island cotton Yuehai 9 and PS-7 was 230 and 31 times higher than that in the roots, respectively (**Figure S9**).

QRT-PCR results of *GbaGELP142D* in island cotton showed that the relative expression of *GbaGELP142D* in the roots and leaves of Yuehai 9 and PS-7 reached a significant difference at 24 h and 4 h under short-term NaCl stress, respectively; however, under long-term NaCl stress, the relative expression of *GbaGELP142D* in the leaves of Yuehai 9 and PS-7 were both higher than those in the roots. This showed that *GbaGELP142D* played an important role in the response of the island cotton Yuehai 9 and PS-7 to salt stress. Therefore, *GbaGELP142D* was selected for overexpression in *Arabidopsis* to verify its salt tolerance further.

### Overexpression of *GbaGELP142D* enhanced salt tolerance in transgenic *Arabidopsis*

**Figure 7:**
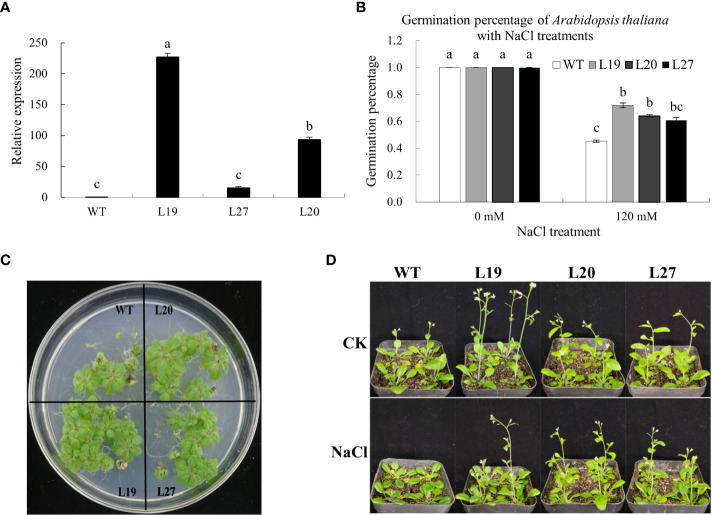
Identification of salt tolerance of *GbaGELP142D* overexpressing *Arabidopsis thaliana*. **(A)** qRT-PCR analysis of *GbaGELP142D* expressing in the wild-types and transgenic *Arabidopsis thaliana* plants. Different letters on the bar graph show a significant difference (P < 0.05). **(B)** Germination percentage of the wild-types and transgenic *GbaGELP142D Arabidopsis thaliana* with different NaCl treatments. **(C)** The phenotype of the seedling of the overexpression lines of the *GbaGELP142D* gene (L19, L20 and L27) and WT under salt stress. **(D)** The phenotype of wild-type plants and transgenic *GbaGELP142D* plants after NaCl treatments.

To study whether the *GbaGELP142D* gene could confer salt tolerance in transgenic plants, we used wild-type and transgenic *Arabidopsis* plants under salt stress for the assay. Three independent T3 *GbaGELP142D* overexpression lines (L19, L20, and L27) were selected by qRT-PCR to examine salinity resistance (**Figure 7A**).

The germination percentages of the wild-types and three transgenic lines of L19, L20, and L27 were all 100% on MS plates containing 0 mM NaCl; however, the germination percentages of three transgenic lines of L19, L20, and L27 were all higher than that of the wild-types on MS plates containing 120 mM NaCl, among them L19 had the highest germination rate (**Figure 7B**). Three transgenic lines (L19, L20, and L27) showed significantly better growth than the wild-type plants 30 days after sowing on MS plates containing 120 mM NaCl (**Figure 7C**). Seven days after the wild-type and three transgenic lines of L19, L20, and L27 were irrigated with 0 mM NaCl and 120 mM NaCl, respectively, compared with the three transgenic lines, the growth of the wild type was significantly inhibited, with some leaves turning yellow, bolting late. The plant height was lower than that of the transgenic lines (**Figure 7D**).

**Figure 8:**
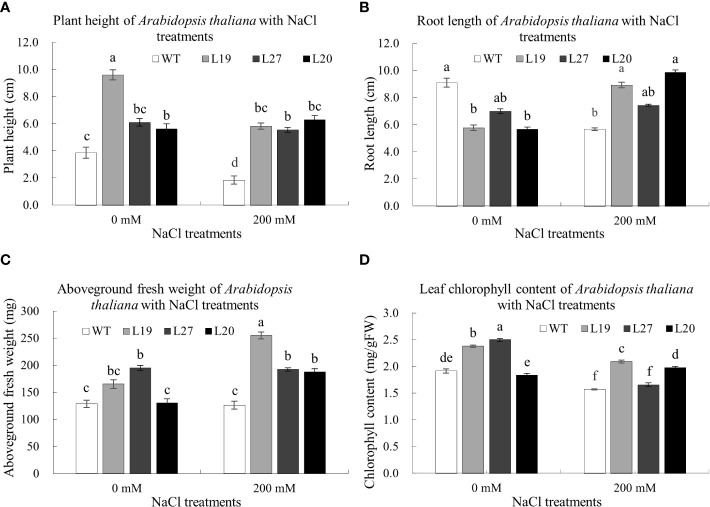
Determination of biological indicators of salt tolerance of *GbaGELP142D* overexpressing *Arabidopsis thaliana*. **(A)** plant height of wild-type plants and transgenic *GbaGELP142D* plants after NaCl treatments. **(B)** root length of wild-type plants and transgenic *GbaGELP142D* plants after NaCl treatments. **(C)** aboveground fresh weight of wild-type plants and transgenic *GbaGELP142D* plants after NaCl treatments. **(D)** leaf chlorophyll content of wild-type plants and transgenic *GbaGELP142D* plants after NaCl treatments. Different letters on the bar graph show a significant difference (P<0.05).

It could be seen from **Figure 8A**, the plant height of transgenic lines was higher than that of the wild-type; compared with the control, the plant height of the wild-type and two transgenic lines, L19 and L27, was lower than that of the control after being treated with 120 mM NaCl for seven days, of which the wild-type was the most reduced by 52.07%; however, the plant height of transgenic line L20 was higher than that of the control. In terms of root length, compared with the control, the root length of the wild type was significantly shorter than that of the control after seven days of 120 mM NaCl treatment, with a reduction of 37.69%, whereas the root lengths of the transgenic lines L19, L20, and L27 were all longer than that of the control, of which L20 had the largest increase, with an increase of 74.07% (**Figure 8B**). In **Figure 8C**, the aboveground fresh weight of the transgenic strain was greater than that of the wild-type; compared with the control, the aboveground fresh weight of wild-type and transgenic line L27 was lower than that of the control after being treated with 120 mM NaCl for seven days, while the aboveground fresh weight of transgenic lines L19 and L20 was higher than that of the control. Among them, the aboveground fresh weight of L19 increased the most, by 54.55%. In terms of chlorophyll content, compared with the control, the chlorophyll content of the wild-type and two transgenic lines L19 and L27 was lower than that of the control after being treated with 120 mM NaCl for seven days, in which the chlorophyll content of the wild-type was reduced by 17.8%; however, the chlorophyll content of transgenic line L20 was higher than that of the control, 8.2% higher than that of the control (**Figure 8D**). Together, these results showed that *GbaGELP142D* transgenic lines had enhanced tolerance to salt stress.

### Molecular mechanism of *GbaGELP142D* enhanced salt tolerance

To analyse the molecular mechanism of salt tolerance, molecular docking and molecular dynamics simulations were performed in this study. After docking, 236 candidate molecules with docking above 0 were identified, and these molecules were fatty acids. The top five molecules were 7E,11E-tetradecadiene-5,9-diynoic acid, methyl 10-acetoxy-8,9-epoxy-2Z-decen-4,6-diynoate, pentadecatri-6,8,10-ynoic acid, deca-2,4,6-triynedioic acid, and 2E,4E,6Z,8Z-decatetraenedioic acid. Analysis of the non-bonded interactions showed that the best compounds interacted with the 7E,11E-tetradecadiene-5,9-diynoic acid-formed salt bridge with His340, a carbon-hydrogen bond with Ser34, and an alkyl with Leu341. Molecular dynamics simulations were used to analyse the conformational changes in the binding pocket. High root-mean-square fluctuation (RMSF) values suggest increased flexibility, whereas low RMSF values reflect decreased flexibility. RMSF analysis of the best compounds revealed that the amino acid residues of the binding pocket showed a flexible conformation. Based on the analysis mentioned above, we presumed that *GbaGELP142D* modulates lipid metabolism by regulating multiple lipid molecules to improve salt resistance.

## Discussion

GELPs and their hydrolysing products play multiple roles in plant growth and development as well as in responses to biotic and abiotic stresses (Shen et al., 2022). In recent years, GDSL lipase has received considerable attention from plant scientists owing to its many biological functions. A large number of *GELP* genes are present in plants. Different numbers (65–194) of GDSL genes have been reported in different plants (Chepyshko et al., 2012; Dong et al., 2016; Lai et al., 2017; Cao et al., 2018; An et al., 2019; Li et al., 2019; Ni et al., 2020; Su et al., 2020; Lv et al., 2021; Ren et al., 2021; Zhang et al., 2021; Jiao et al., 2022; Sun et al., 2022; Zhang et al., 2022). Therefore, it was not difficult to find that allotetraploid cotton harboured a larger number of *GELP* genes. Moreover, we found that the number of *GELP* genes derived from the Dt sub-genome of the five allotetraploid cotton species was greater than that of the *GELP* genes of the D_5_ genome. This indicates that the *GELP* genes of the Dt sub-genome play an important role in the adaptation of cotton to environmental fluctuations after the formation of allotetraploids.

Kong et al. (2020) reported that GELPs existed as early as in the charophyte *Penium margaritaceum*, the last common ancestor of the Zygnematophyceae and the land plants. As an ancient lycophyte, *Selaginella moellendorffi* diverged shortly after land plants evolved into vascular plants and GELPs expanded significantly (Vujaklija et al., 2016). Shen et al. proposed that GELPs play an important role in the colonisation of land by plants (Shen et al., 2022); in light of this GELPs are found in all land plants (Smyth, 2017). Based on these studies, we speculated that the reason for the large number of *GELP* genes is related to their unique functions in species.

### *GELP* genes evolution in *Gossypium*

In the present study, a similar topology was displayed in a phylogenetic tree constructed using GELPs from *A. thaliana* and *O. sativa* (Chepyshko et al., 2012; Lai et al., 2017). However, there were three GELPs (AtGELP62, AtGELP85, and AtGELP86) divided into clade III, compared with those in clade IV, in *A. thaliana*. This may be due to the different methods used to generate the phylogenetic trees. In addition, the unrooted phylogenetic tree, which included 1502 GELPs from 10 species, had 16 subclades. A total of 140 conserved motifs were found in the 1502 GELPs. A total of 148 orthologous gene clusters were obtained, and 102 orthologous gene clusters were solely composed of genes found in cotton species. Orthologous genes also illustrated that the *GELP* gene family diverged across different species during evolution. The 3D structures of the 16 GELPs from each subclade showed that the GELP family has high structural diversity. Molecular docking results revealed that the GELPs also had substrate diversity. The basic physicochemical properties of the 1502 GELP proteins vary widely. The above-mentioned results show that *Gossypium* species have a high diversity of GELPs.

Gene duplication analysis indicated that *GELP* genes have different rates of evolution among the five allotetraploids. The genomic diversification of the five allotetraploid species, which have a monophyletic origin, was accompanied by biogeographic radiation to the Galapagos Islands (Gda), the Hawaiian Islands (Gto), South America (north-eastern Brazil) (Gmu), Central and South America, the Caribbean, and the Pacific (Ghi and Gba) (Chen et al., 2020b). These results reflect the role of *GELP* genes in the ecological adaptation to natural environments. Similar studies have suggested that different sub-genomes experience different rates of protein-sequence evolution in cotton (Sharbrough et al., 2022). Therefore, we determined that segmental duplication and differences in evolutionary rates were the leading causes of the increase in the number and diversity of *GELP* genes during evolution for ecological adaptation.

### The molecular mechanisms of *GELPs* functional diversity

The promoters of the *GELP* gene family contain largely *cis*-acting motifs related to plant growth and development, hormones, biotic stress, and abiotic stress. Subcellular localisation prediction showed that GELP genes were present in all parts of the cell. More than 64% of GELPs contain signal peptides and >29% of GELPs contain one potential transmembrane domain. The expression patterns of a large number of *GELP* genes were specifically expressed in different tissues or developmental stages and were differentially expressed under different stresses (**Figure 6**). Furthermore, multiple *GELP* genes can be induced by various abiotic (cold, hot, salt, and drought) and biotic (cotton aphid attack) stresses. It has previously been shown that GELPs exert essential functions in many physiological and biochemical processes (Akoh et al., 2004; Ding et al., 2019; Shen et al., 2022). Therefore, we found that cotton GELPs play a significant role in plant responses to various stressors during plant growth and development.

Although GDSL lipase genes have been isolated from several plants, our knowledge of plant GDSL lipases, from sequences to functional mechanisms, is limited (Ding et al., 2019). The characteristics and functions of plant GDSL lipases have been identified and investigated in approximately 20 plant species. However, studies on the molecular mechanisms underlying functional diversity in plant GELPs have yet to be reported. Studying lipase activity is one of the most important molecular mechanisms underlying *GELPs* functional diversity. *RMS2*, which leads to significant changes in the content of 16 lipid components, may serve as a key node in the male fertility regulatory network in rice (Zhao et al., 2020c). Zhao et al. found that MHZ11 impairs receptor-OsCTR2 interactions and OsCTR2 phosphorylation to trigger ethylene signalling by reducing sterol levels (Zhao et al., 2020a). The thioesterase activity of OSP1, which is essential for wax biosynthesis and stomatal function, was tested using *p*-nitrophenyl acetate and *p*-nitrophenyl butyrate (Tang et al., 2020). *ZmMs30* modulates pollen exine formation and anther cuticle development *via* an aliphatic metabolic pathway (An et al., 2019). Currently, only one study has reported the biochemical characterisation of rice GELPs with a known natural substrate (Zhang et al., 2017; Zhao et al., 2020c). To date, the hydrolase activity of plant GELPs has been assessed using common substrates and metabolomics, but there are more appropriate methods than these two approaches.

The reasons for the difficulty in determining a suitable substrate are as follows: (1) Unlike other lipases, GDSL lipases have a flexible active site in their structure that leads to the binding of different substrates, resulting in more diverse enzyme activities (Huang et al., 2001; Akoh et al., 2004; Ding et al., 2019). (2) Homology modelling is an efficient approach for predicting 3D structures. The crystal structures of GDSL lipase proteins from some microbes have been previously determined in PDB. However, 3D protein structures of plant GDSL lipases have yet to be reported (Ding et al., 2019). GDSL enzymes exhibit a low overall sequence similarity (Akoh et al., 2004; Vujaklija et al., 2016). Nevertheless, lower sequence similarity is a significant limitation of this method. In this study, the BLAST against the PDB showed that out of 1502 GELP members, only GarGELP71A had the highest protein sequence identity with 3MIL from *Saccharomyces cerevisiae* (35.8%). Recently, as a novel machine learning approach, AlphaFold2 has been used to predict protein structure based on the amino acid sequence and has made landmark advances in protein structure prediction (Jumper et al., 2021). (3) Many plant-derived proteins fail to express and fold properly in prokaryotic expression systems; thus, their activities and substrate specificities can be affected (Hua, 2008; Li et al., 2014; Ding et al., 2019). Therefore, for GELP lipases in plants, identifying the natural substrates and studying their biochemical functions, became a great challenge *in vitro* and *in vivo* (Ding et al., 2019; Tang et al., 2020; Zhao et al., 2020a; Shen et al., 2022).

To overcome this challenge, we used molecular simulation techniques to analyse the 3D structure of GELPs and assess the properties and conformational changes of the binding pocket based on the phylogeny and genetic analysis of the *GELP* gene family in cotton. The 3D structures of the 16 GELP lipase proteins were generated using AlphaFold2 and homology modelling. The results highlighted notable differences in the structures of the 16 GELPs. Similar results were reported by Jiao et al. (2022), in which the spatial structures of CilGDSL in pecans were also different. Our molecular docking experiment revealed that the number of docking ligands varied greatly between individual proteins. For example, the number of ligands docking the GhiGELP172D protein was 0, whereas GdaGELP59A had 490 ligands docking. This suggests that the properties of the binding pocket were significantly different due to the differences in amino acid residues outside the binding site. Cavities with similar functionalities are often conserved across protein families (Stank et al., 2016). Our results suggest that binding pockets are not conserved across GELPs families. This could be the reason for the diversity of GELP functions and particularity of GELPs.

The GELPs in subclade IIIC had unique structures. For example, typical GDSL were located in motifs 36 and 71 in block I. The 3D structures and substrates showed larger differences compared to the other subclades. Therefore, it can be inferred that GELPs in subclade IIIC have special functions.

GDSL lipases have a flexible active site in their structure that leads to the binding of different substrates, resulting in more diverse enzyme activities. For example, because of the flexibility of the substrate-binding pocket of TEP-I, it can bind different substrates in optimal conformations for catalysis, making it an extremely versatile enzyme, such as a lysophospholipase, arylesterase, protease, and thioesterase (Huang et al., 2001; Akoh et al., 2004). The binding pocket of GbaGELP142D also exhibited a flexible conformation. Therefore, the intrinsic dynamic properties of the binding pockets of GELPs may be one of the reasons for their diverse functions.

Therefore, to characterise the binding pocket to uncover the diverse functions of GELPs, it was found that the properties of the binding pockets include not only the volume, shape, hydrophobicity, electrostatics, and chemical fragment interactions, but also dynamic properties such as stability, continuity, and correlation (Schreyer and Blundell, 2009; Henrich et al., 2010; Stank et al., 2016; Chen et al., 2019). The properties of ligand-binding pockets should be helpful in understanding the functional mechanisms of GELPs.

## Data availability statement

The datasets presented in this study can be found in online repositories. The names of the repository/repositories and accession number(s) can be found in the article/**Supplementary Material**.

## Author contributions

JW, HZ, YQ, JH planned and designed the study. JW and HZ performed experiments. JW wrote the manuscript. HZ and PY provided principal funding. We have read, edited, and approved the manuscript for publication. All authors contributed to the article and approved the submitted version.

## Supplementary material

Supplementary Table 1 The primers used for qRT-PCR and gene cloning.

Supplementary Table 2 The basic physicochemical properties, signal peptides, predicted transmembrane structure, and chromosome location of 1502 GELPs.

Supplementary Table 3 A list of gene IDs on the phylogenetic tree.

Supplementary Table 4 The motif compositions of 1502 GELPs on the phylogenetic tree in cotton.

Supplementary Table 5 The Ka/Ks values of the duplicate gene pairs among 10 cotton species.

Supplementary Table 6 The evolution rates of GELPs among five allotetraploids.

Supplementary Table 7 The orthologous gene clusters among the ten cotton species and *Arabidopsis thaliana*.

Supplementary Table 8 The templates of homology modeling of 16 GELPs.

Supplementary Table 9 The substrate specificity of 15 GELPs.

Supplementary Table 10 The FPKM values of the heatmaps.
