# Supplementary material for: The binding pocket properties were fundamental to functional diversification of the GDSL-type esterases/lipases gene family in cotton
Source: Front Plant Sci. 2023 Jan 18;13:1099673. doi: 10.3389/fpls.2022.1099673 (PMC9889996; doi:10.3389/fpls.2022.1099673)
Supplement: Supplementary file 1 [file Presentation_1.zip › Fig S.docx]

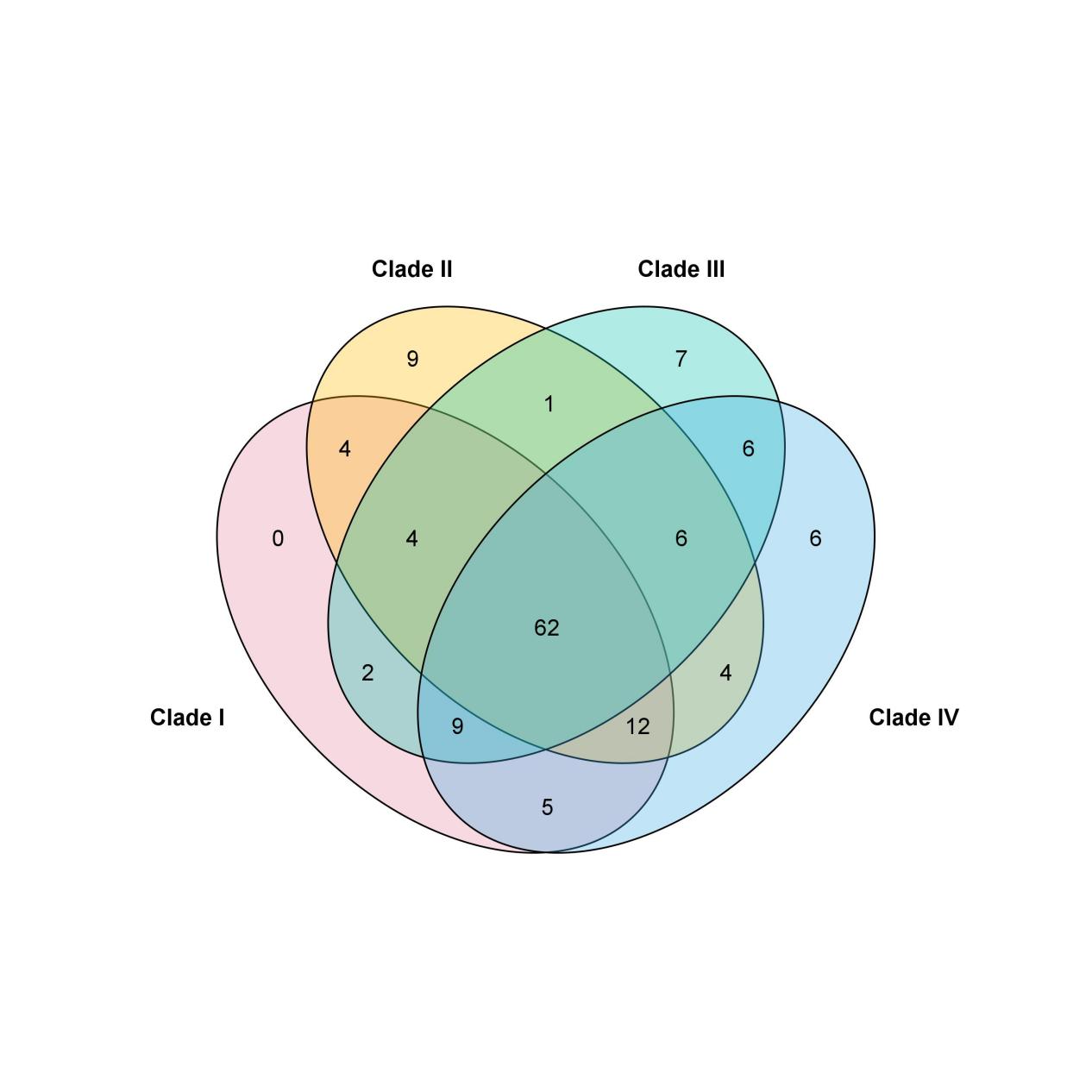


**Figure S1** The Venn chart of the conserved motif analysis according to the phylogenetic tree.


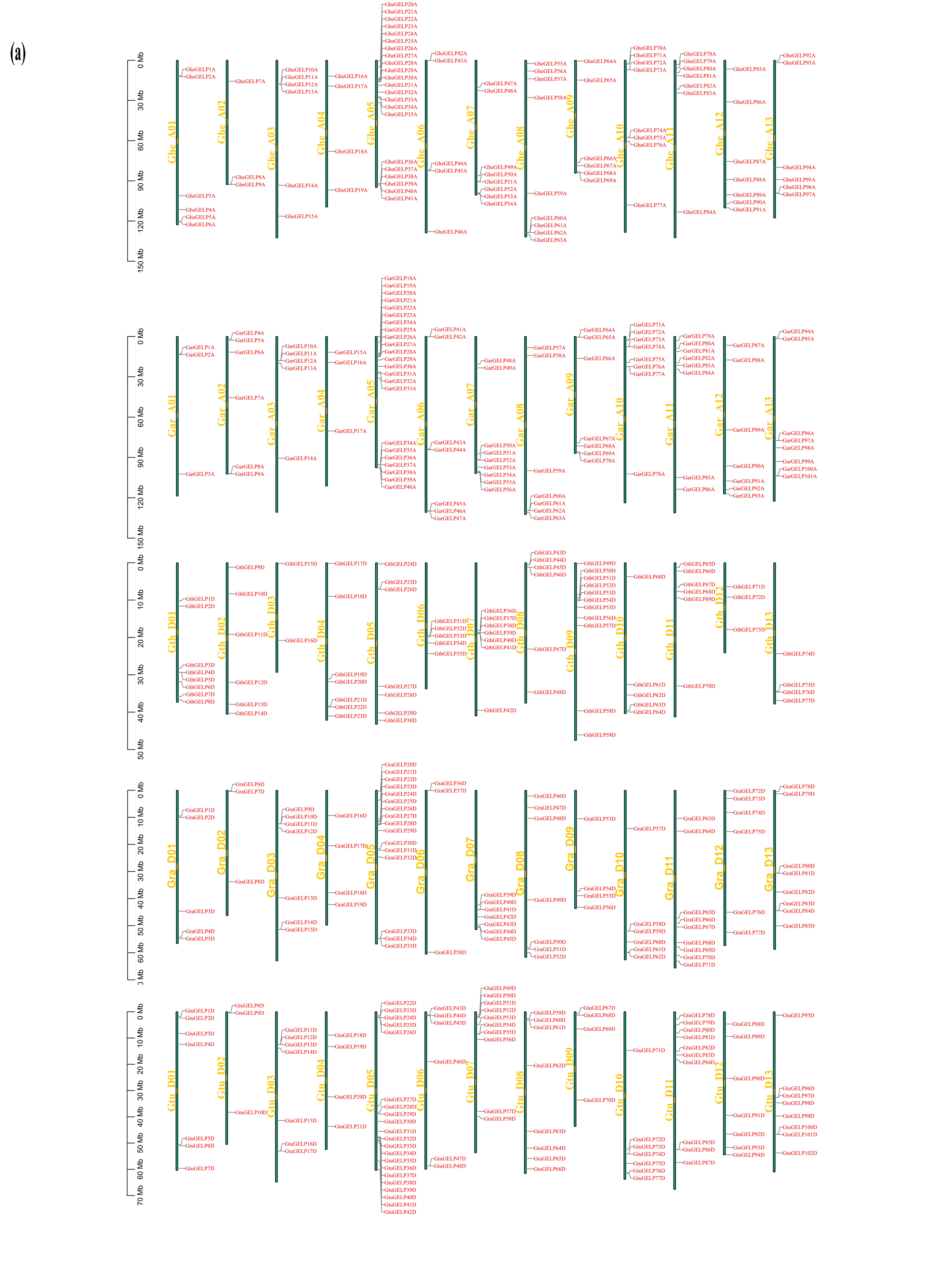


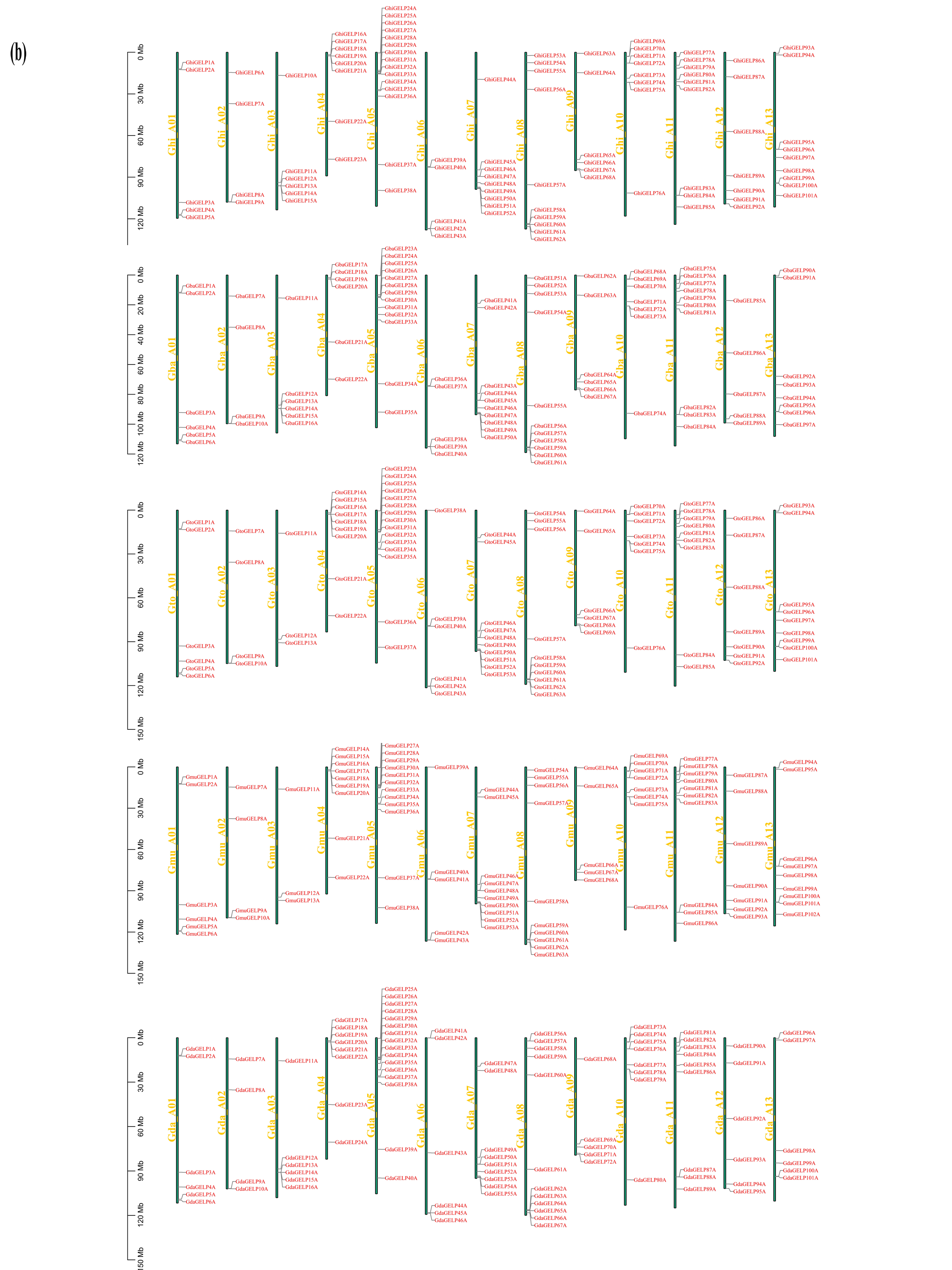


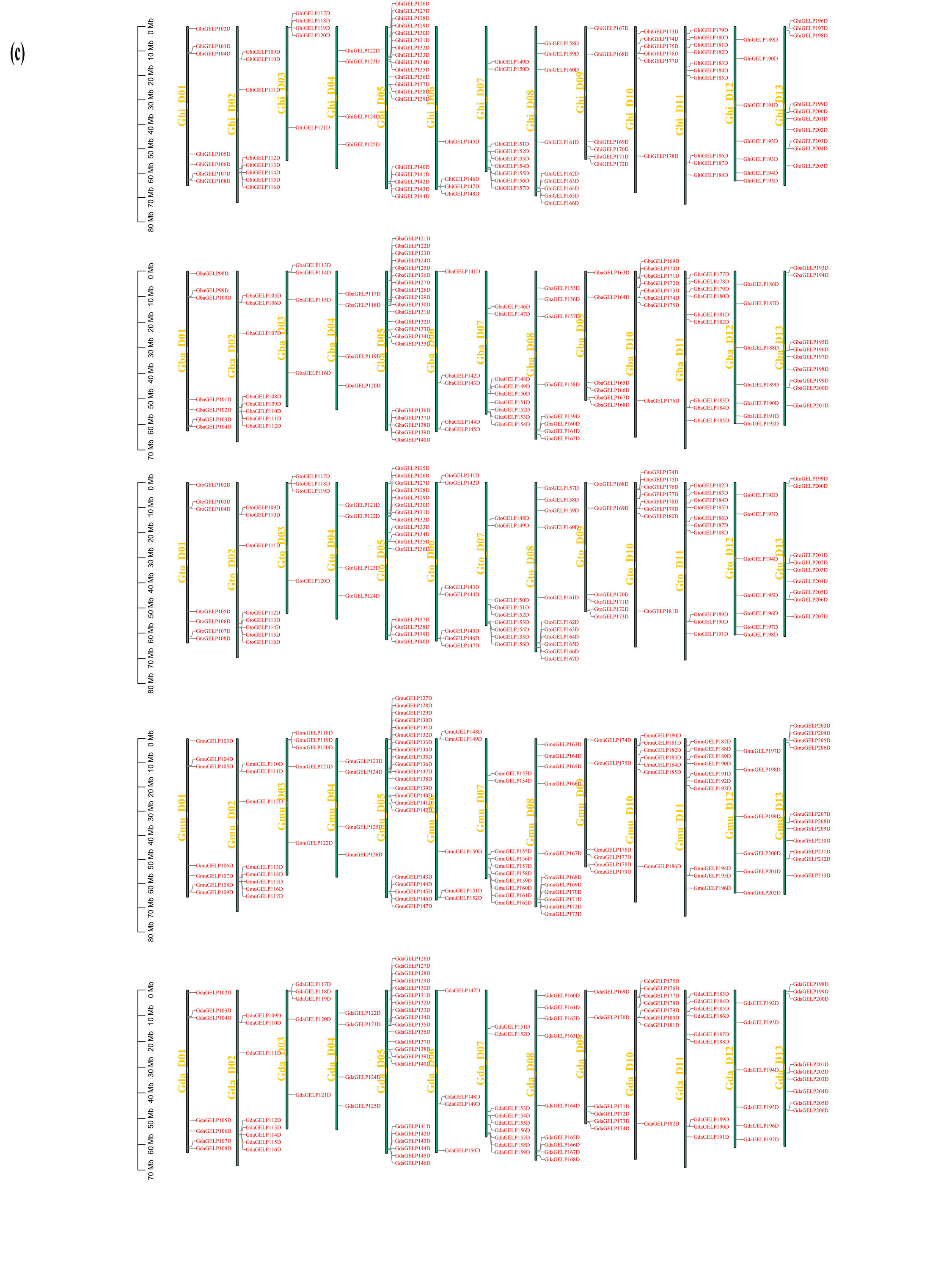


**Figure S2** Chromosomal positions of GELPs from 10 cotton species with gene IDs shown on the right side. The vertical bar on the left side represents the position of the gene and length of the chromosome.


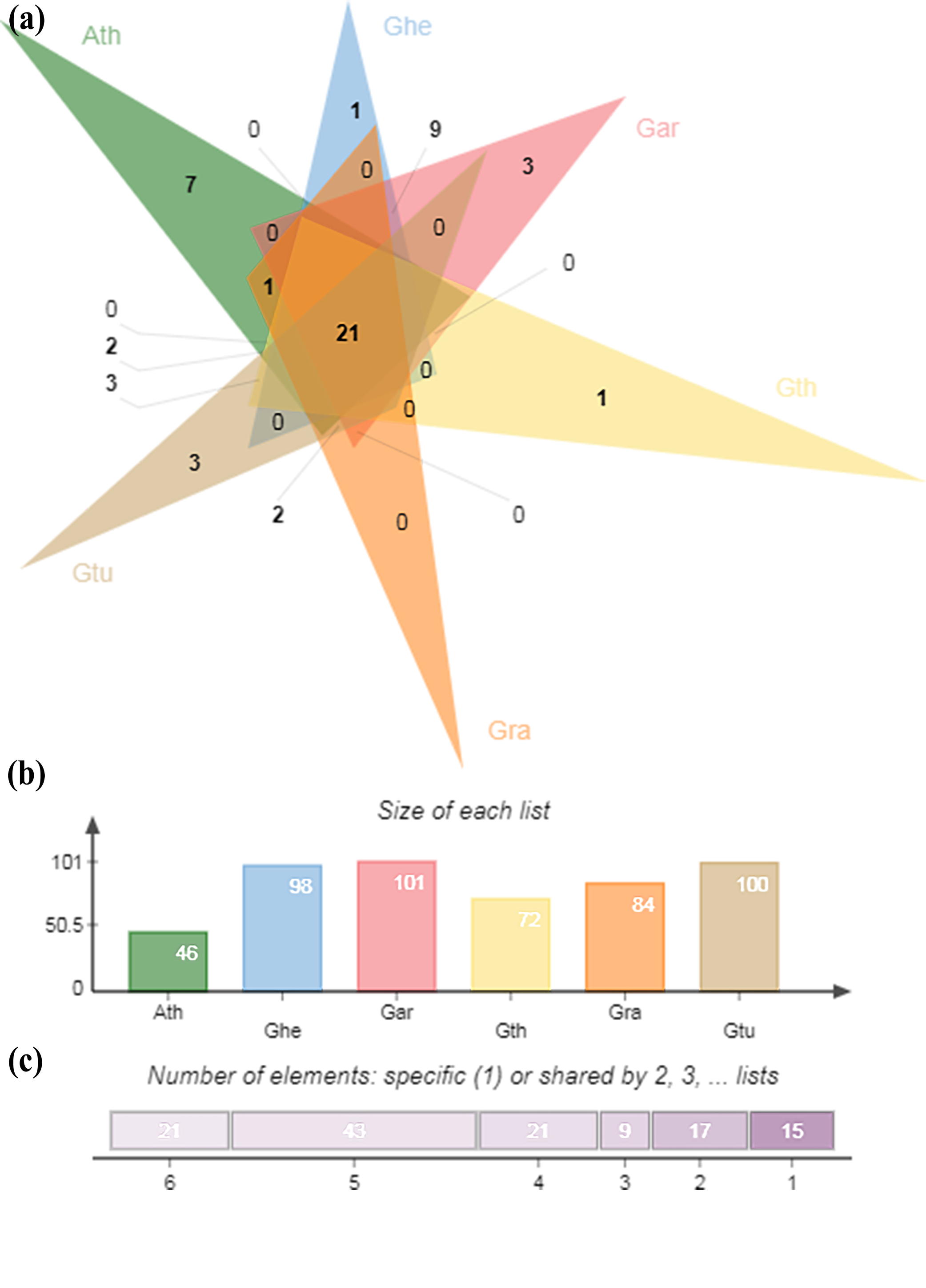


**Figure S3** Analysis of the clustering of orthologous genes between (a) *Gossypium herbaceum*, *Gossypium arboreum*, *Gossypium thurberi*, *Gossypium raimondii, Gossypium turneri* and *Arabidopsis thaliana*. (b) The size of each list, and (c) number of elements shared.


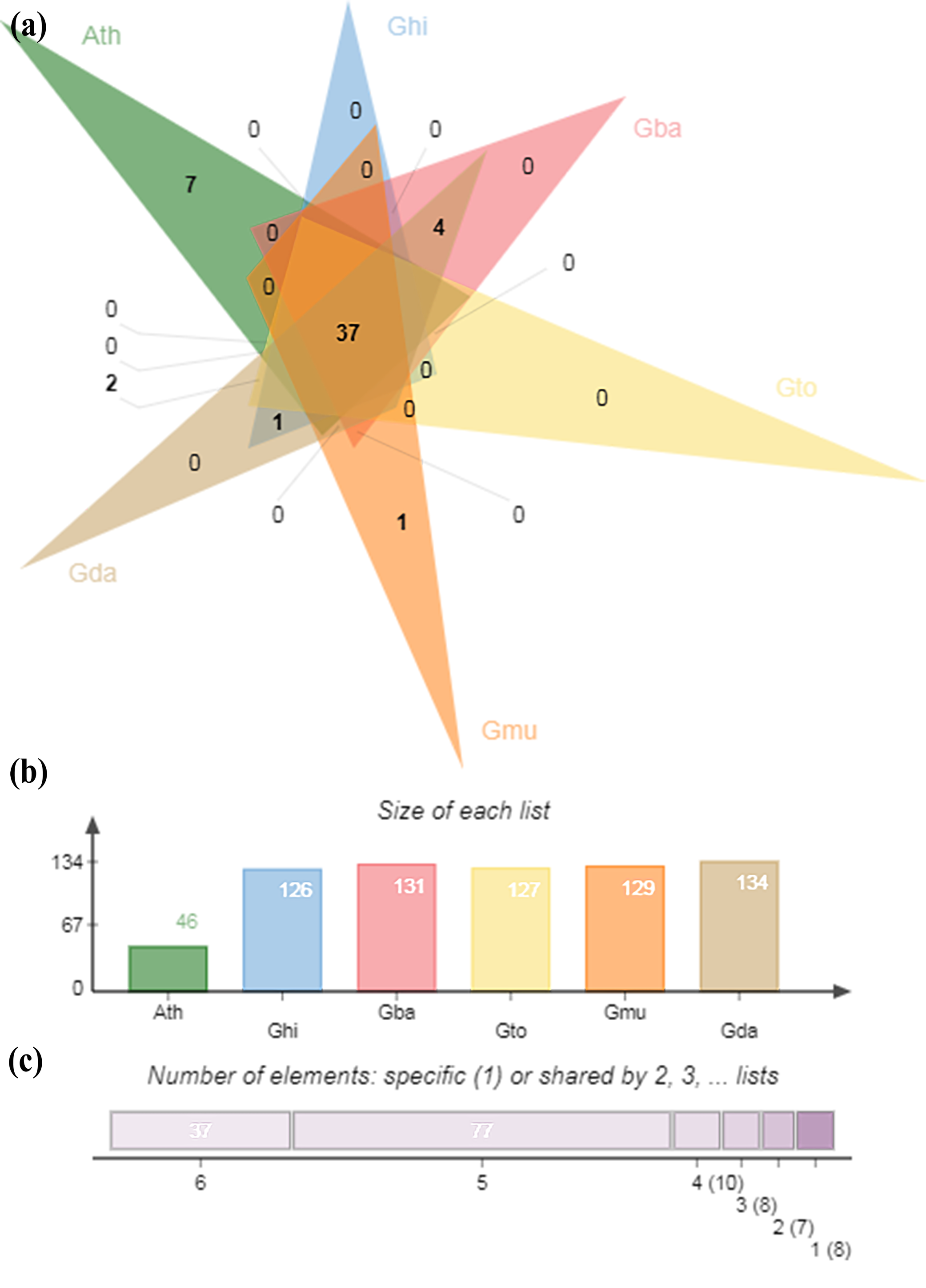


**Figure S4** Clustering of orthologous genes analysis between (a) *Gossypium hirsutum*, *Gossypium barbadense*, *Gossypium tomentosum*, *Gossypium mustelinum*, *Gossypium darwinii* and *Arabidopsis thaliana*. (b) The size of each list, and (c) number of elements shared.


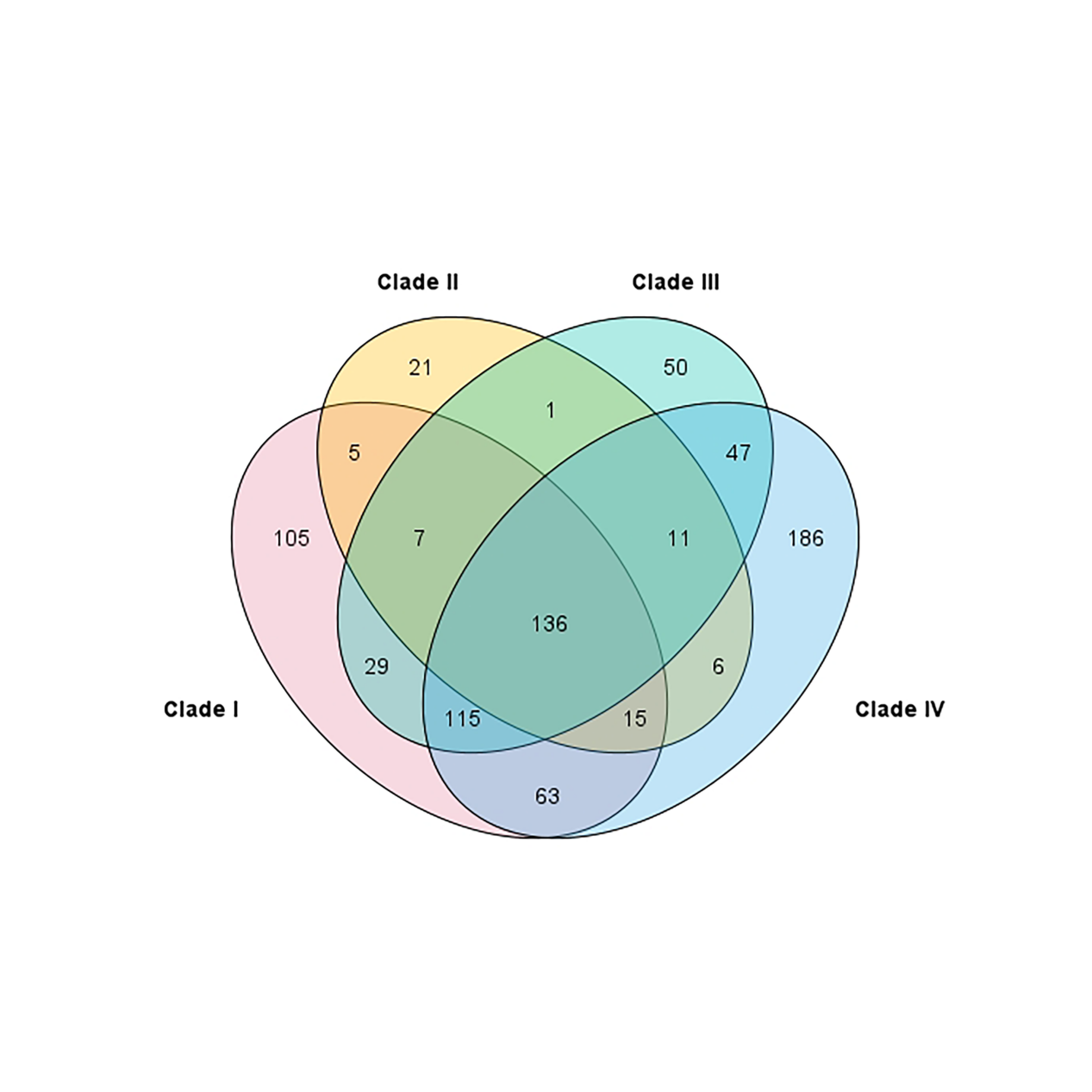


**Figure S5** The Venn chart of the unique lipid analysis according to the phylogenetic tree.


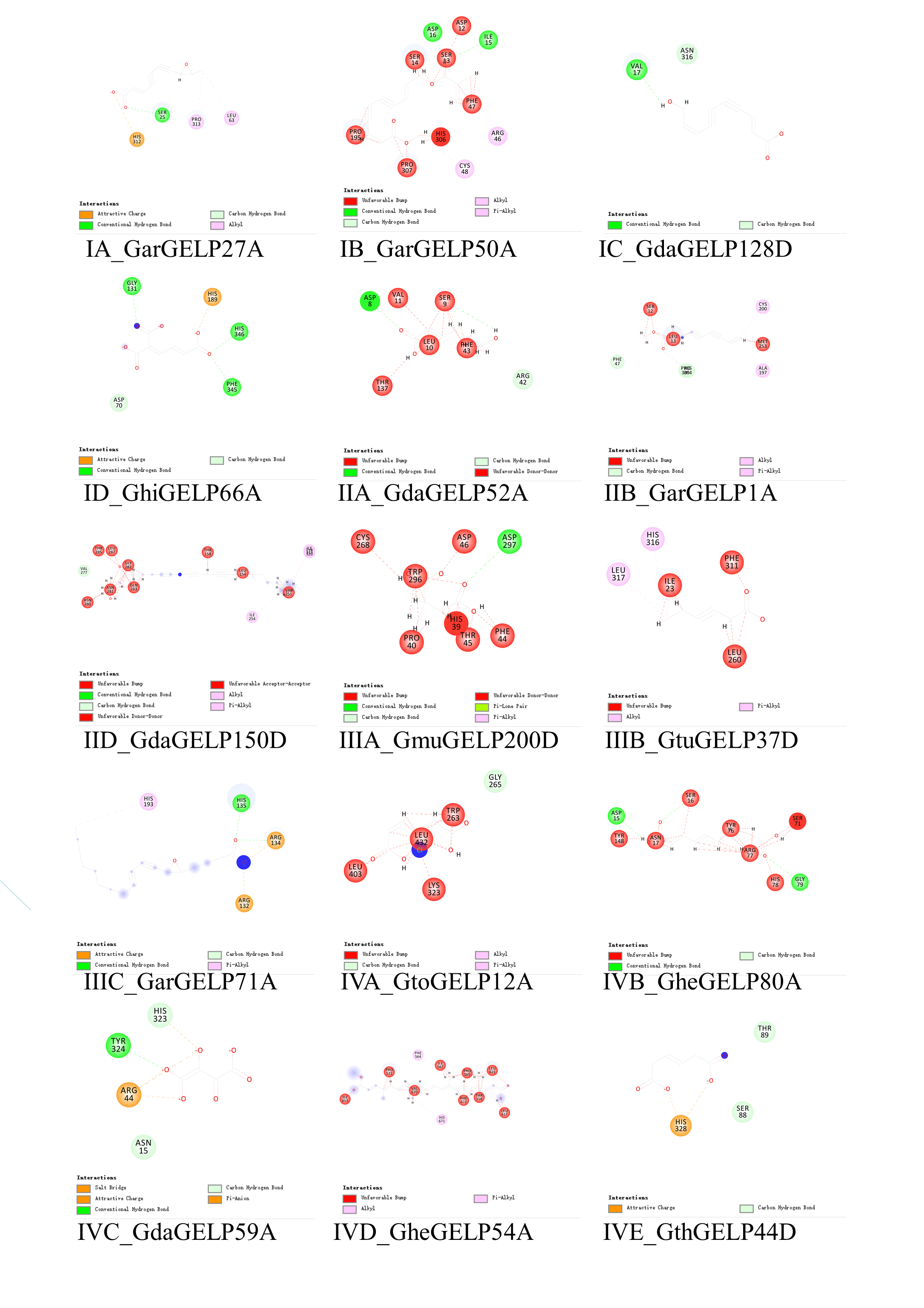


**Figure S6** Non-bounded interaction force between the GELPs and the ligand which had maximum -CDOCKER_INTERACTION_ENERGY value.


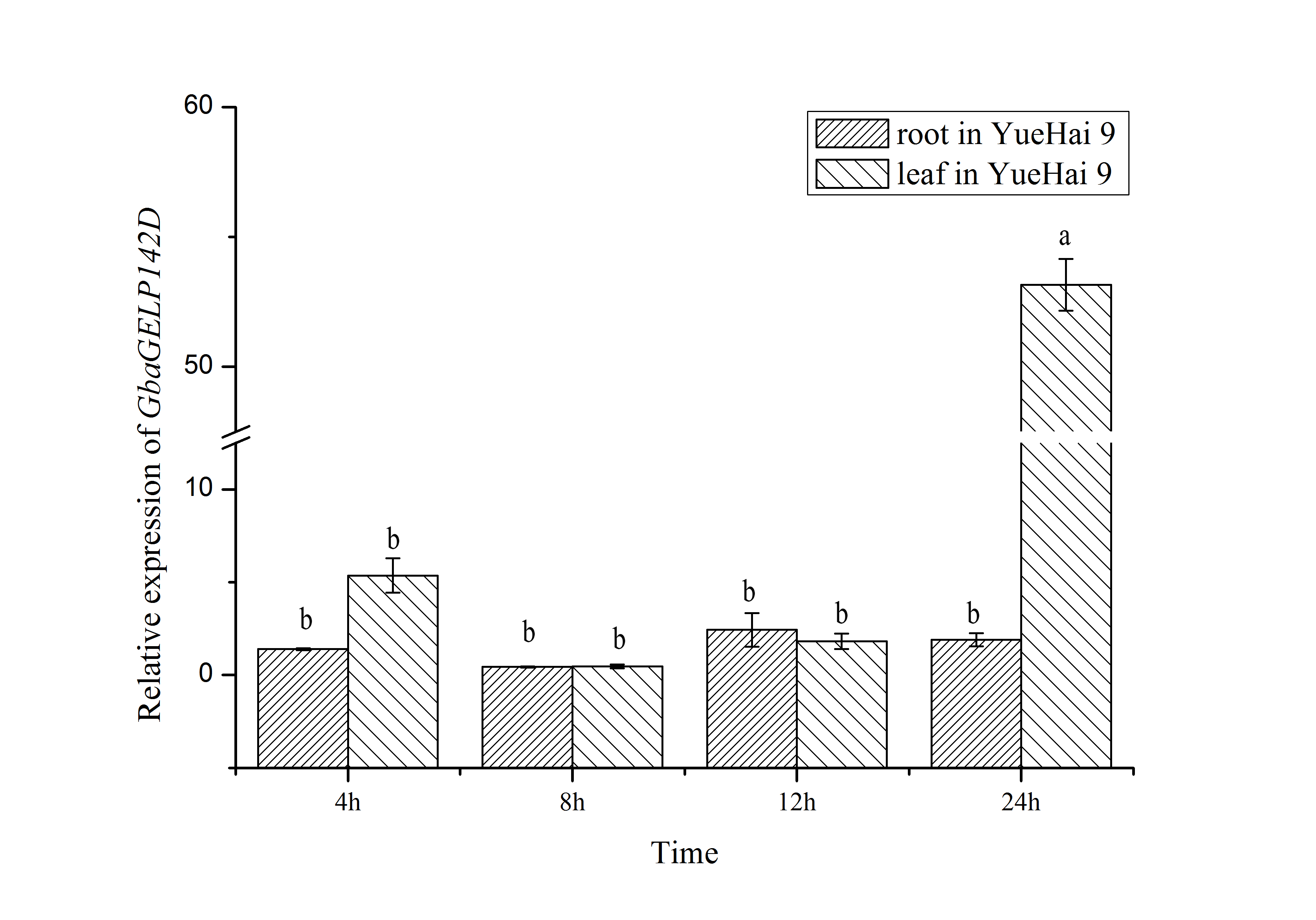


**Figure S7** QRT-PCR analysis of *GbaGELP142D* in Yuehai 9 under NaCl short-term stress. Different letters on the bar graph show significant difference (P<0.05).


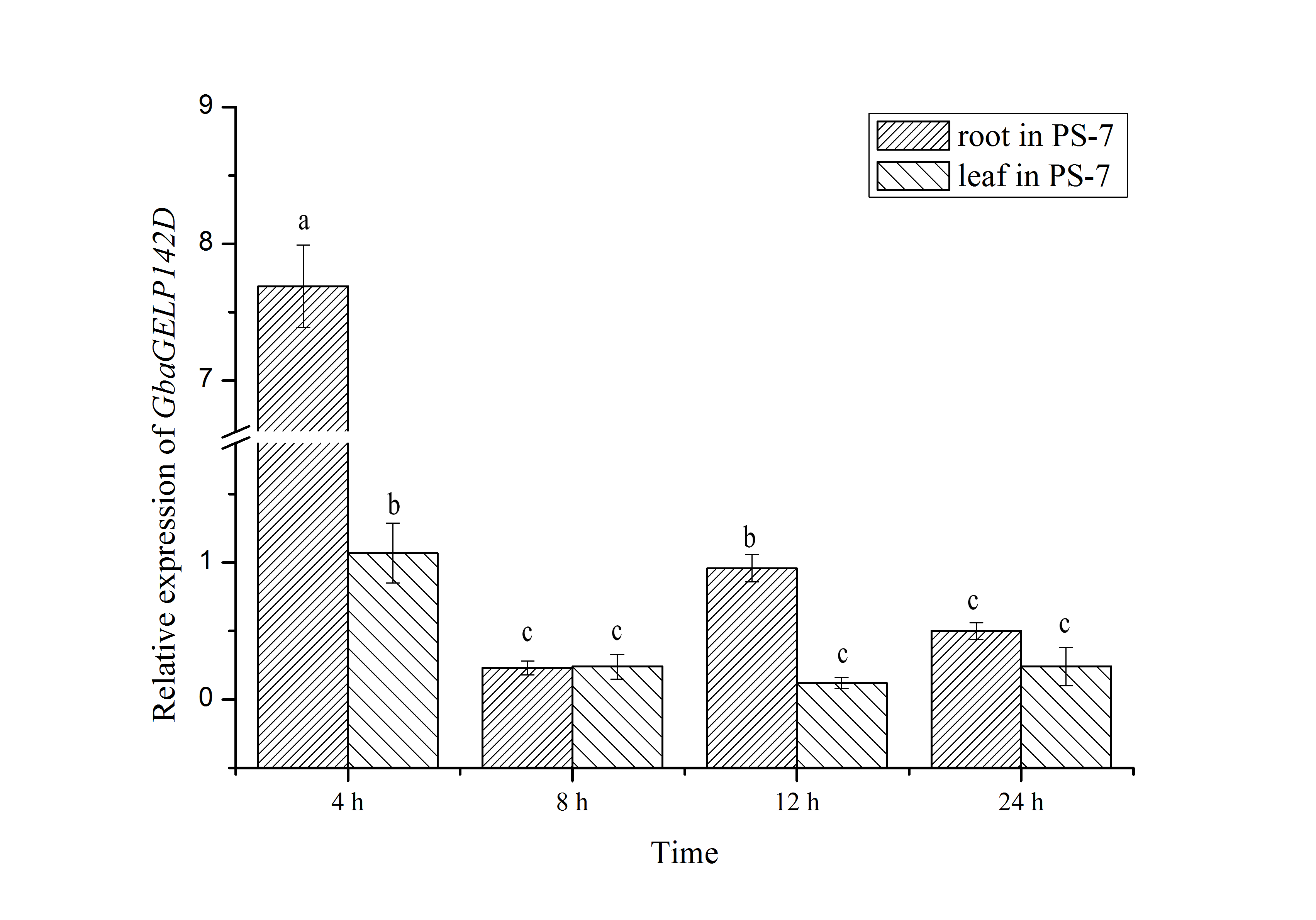


**Figure S8** QRT-PCR analysis of *GbaGELP142D* in PS-7 under NaCl short-term stress. Different letters on the bar graph show significant difference (P<0.05).


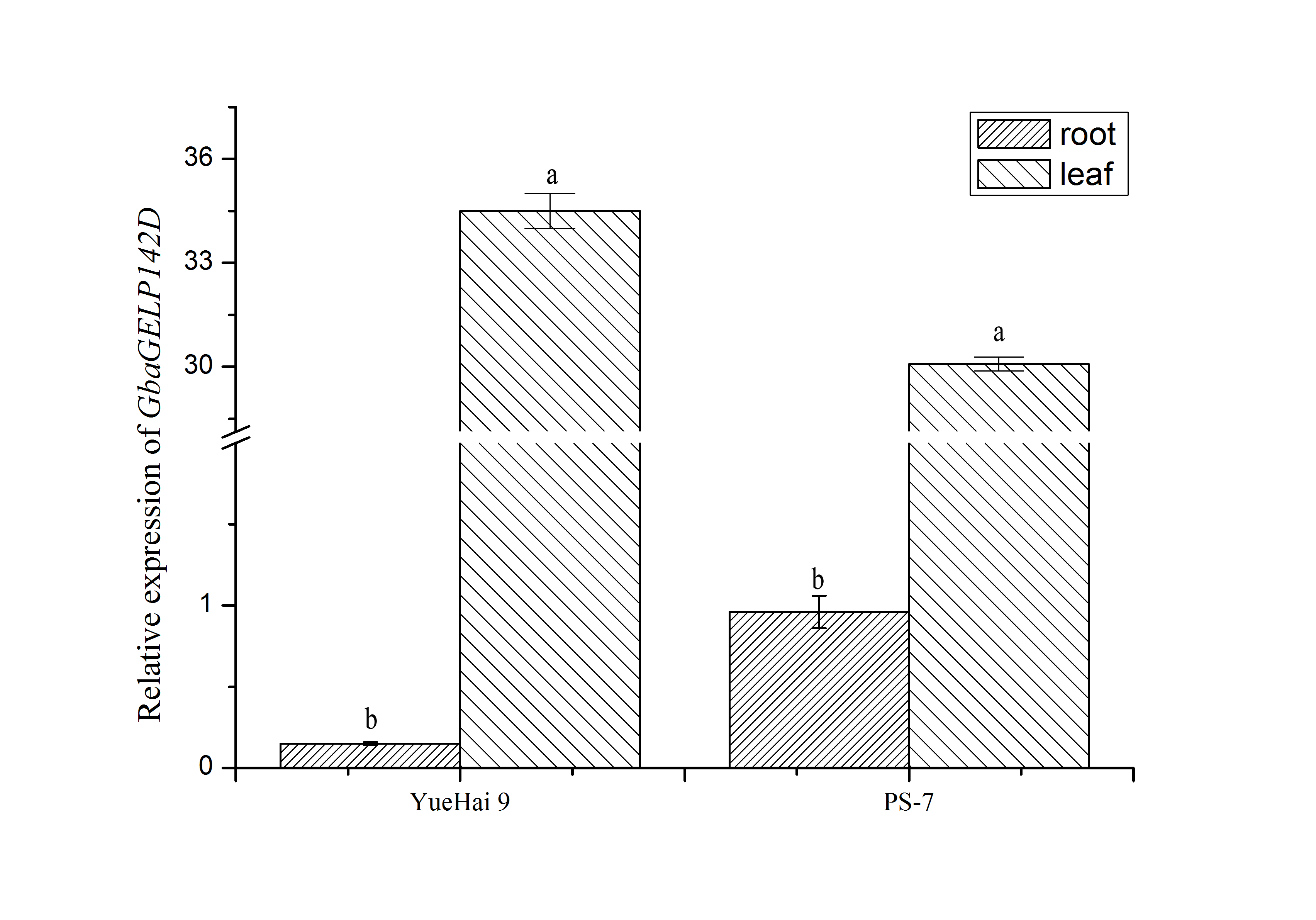


**Figure S9** QRT-PCR analysis of *GbaGELP142D* in Yuehai 9 and PS-7 under NaCl long-term stress. Different letters on the bar graph show significant difference (P<0.05).
